# Ubiquitin C-terminal hydrolase37 regulates Tcf7 DNA binding for the activation of Wnt signalling

**DOI:** 10.1038/srep42590

**Published:** 2017-02-15

**Authors:** Wonhee Han, Hyeyoon Lee, Jin-Kwan Han

**Affiliations:** 1 Department of Life Sciences, Pohang University of Science and Technology, 77 Cheongam-Ro, Nam-Gu, Pohang, Gyeongbuk, 37673, Korea

## Abstract

The Tcf/Lef family of transcription factors mediates the Wnt/β-catenin pathway that is involved in a wide range of biological processes, including vertebrate embryogenesis and diverse pathogenesis. Post-translational modifications, including phosphorylation, sumoylation and acetylation, are known to be important for the regulation of Tcf/Lef proteins. However, the importance of ubiquitination and ubiquitin-mediated regulatory mechanisms for Tcf/Lef activity are still unclear. Here, we newly show that ubiquitin C-terminal hydrolase 37 (Uch37), a deubiquitinase, interacts with Tcf7 (formerly named Tcf1) to activate Wnt signalling. Biochemical analyses demonstrated that deubiquitinating activity of Uch37 is not involved in Tcf7 protein stability but is required for the association of Tcf7 to target gene promoter in both *Xenopus* embryo and human liver cancer cells. *In vivo* analyses further revealed that Uch37 functions as a positive regulator of the Wnt/β-catenin pathway downstream of β-catenin stabilization that is required for the expression of ventrolateral mesoderm genes during *Xenopus* gastrulation. Our study provides a new mechanism for chromatin occupancy of Tcf7 and uncovers the physiological significance of Uch37 during early vertebrate development by regulating the Wnt/β-catenin pathway.

The Wnt/β-catenin pathway is highly conserved across species and is involved in various biological processes, including embryonic development^1^. The most important factor of this pivotal signalling is transcriptional activation that is mediated by complex formation between Tcf/Lef family proteins and β-catenin, a transcriptional co-activator. To ensure precise complex formation, Tcf/Lef proteins are thought to be elaborately regulated. Post-translational modifications (PTMs) have been suggested to be an effective regulatory mechanism for prompt and accurate regulation of Tcf/Lef activity without *de novo* protein synthesis^2^. PTMs of Tcf/Lef proteins, including phosphorylation, acetylation, sumoylation and ubiquitination, regulate interactions with transcriptional co-factors, transcriptional activities, DNA binding ability or protein abundance^2^,^3^. Despite the fact that ubiquitination exerts both proteolytic and non-proteolytic regulation on its substrates, only proteolytic regulation of Tcf/Lef proteins has been investigated^3^,^4^, whereas non-proteolytic regulation of Tcf/Lef proteins is largely unknown.

In vertebrate development, four Tcf/Lef proteins are functionally specialized. The distinct roles of vertebrate Tcf/Lef family members have been intensively studied using the *Xenopus* embryo as the most adequate model system to investigate Wnt signalling^5^,^6^,^7^,^8^. Their specialized functions are crucial for mesoderm development during *Xenopus* gastrulation. Zygotically expressed Tcf/Lef proteins specifically regulate mesoderm induction and subsequent mesoderm patterning by mediating zygotic Wnt/β-catenin signalling, which is triggered by ventrally expressed Wnt8^9^,^10^. Tcf7 and Tcf7l1 (formerly named Tcf3) are independently required for mesoderm induction as a transcriptional activator and repressor, respectively, and both Tcf7 and Lef1 mediate mesoderm patterning as transcriptional activators^9^,^10^.

Ubiquitin C-terminal hydrolase 37 (Uch37) is a deubiquitinating enzyme (DUB) that is functionally linked to multiple protein complexes^11^,^12^,^13^. Uch37 associates with the proteasome and removes ubiquitin moieties from target proteins. As a result, the proteins are protected from proteasome-dependent proteolysis^14^,^15^,^16^. Recently, it was suggested that Uch37 also regulates genome integrity and gene transcription in the nucleus. Nuclear Uch37 mediates DNA double-strand breaks (DSBs) repair by stabilizing the nuclear factor related to *κ*B-binding protein (NFRKB), a subunit of the INO80 chromatin remodelling complex^17^. In addition, Uch37 deubiquitinates E2 promoter binding factor 1 (E2F1), promoting transcription of pro-apoptotic and cell cycle-related genes^18^. However, *in vivo* functions of Uch37 and its substrates during vertebrate embryogenesis remain unclear.

Here, we report that Uch37 mediates the deubiquitination of Tcf7 without affecting protein stability. Moreover, we suggest that enzymatic activity of Uch37 is required for DNA binding of Tcf7 in *Xenopus* gastrula embryo and human liver cancer cells. Our *in vivo* analyses reveal that Uch37 acts as a positive regulator of the Wnt/β-catenin pathway by regulating the expression of ventrolateral mesoderm genes during *Xenopus* gastrulation.

## Results

### Phenotypic results of Uch37 knockdown experiments in *Xenopus*

Uch37 is highly conserved across vertebrate species^11^ and remarkably, *Xenopus* Uch37 and its human orthologue are over 95% identical in amino acid sequences (Supplementary Fig. S1). To elucidate the endogenous function of Uch37 in *Xenopus*, we first analysed the spatiotemporal expression of Uch37 transcripts. RT-PCR analysis showed that Uch37 transcripts were expressed in unfertilized eggs through the tadpole stage (Supplementary Fig. S2a). *In situ* hybridization further revealed that Uch37 transcripts maintained strong expression at the animal hemisphere from the four-cell stage to the late blastula stage (Supplementary Fig. S2b). Notably, with the onset of gastrulation, Uch37 transcripts became enriched in the mesodermal region that resides in the marginal zone of the gastrula embryo (Supplementary Fig. S2b). RT-PCR analysis using dissected explants from gastrula embryos consistently showed stronger enrichment of Uch37 transcripts in both the dorsal and ventral mesoderm compared with animal ectoderm (Supplementary Fig. S2c). These results suggest that Uch37 may act dominantly in the mesodermal region of the embryo during *Xenopus* gastrulation.

In order to understand the endogenous function of Uch37 in *Xenopus*, as suggested by its local enrichment in the mesodermal region of the embryo, we knocked down endogenous Uch37 by injecting antisense morpholino oligonucleotides (Uch37 MO) that specifically and efficiently blocked translation of Uch37 protein (Supplementary Fig. S3a). Equivalent injections of Uch37 MO in dorsal and ventral marginal zones (DMZ and VMZ) in four-cell stage embryos showed that ventrally depleted Uch37 caused posterior defects including loss of dorsal fin and abnormal tail shape (Supplementary Fig. S3b left, c, Table S1), whereas dorsally depleted Uch37 caused anterior truncation at the tadpole stage (Supplementary Fig. S3b right, d, Table S1). The severity of defects in Uch37 morphants was ameliorated by the addition of the MO-resistant form of Uch37 mRNA (Re.Uch37), confirming specificity of the MO effects (Supplementary Fig. S3c,d, Table S1). Collectively, these results indicate that Uch37 is required for *Xenopus* mesoderm development.

### Uch37 is required for Wnt signalling downstream of β-catenin

**Figure 1:**
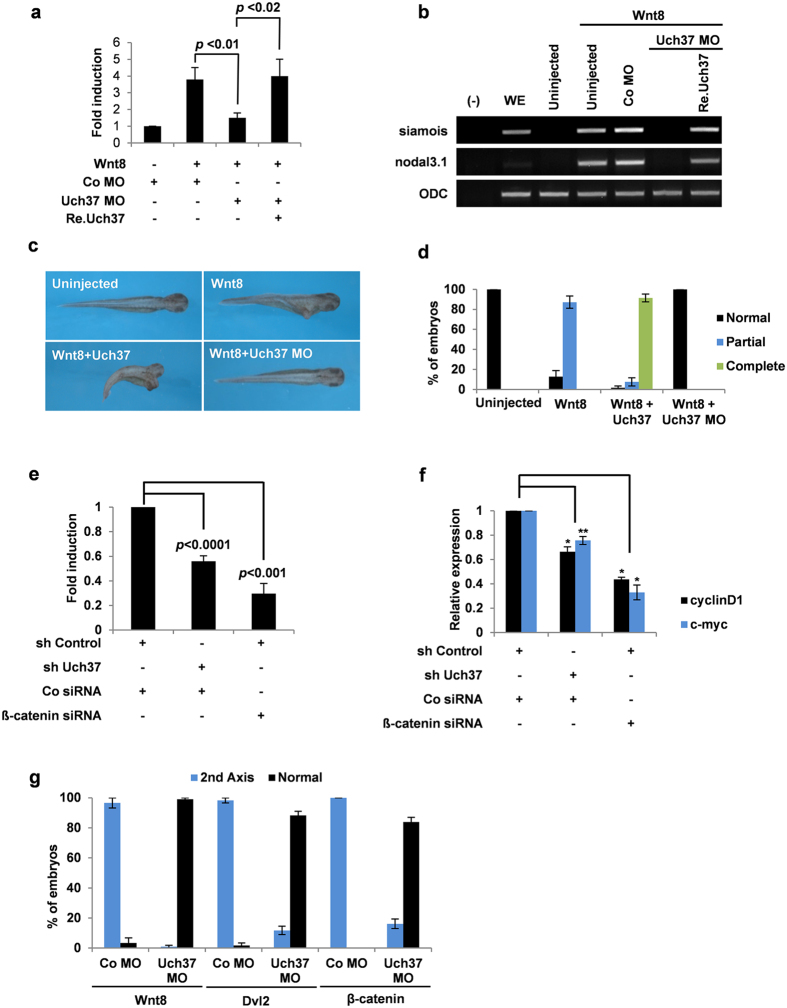
Uch37 positively regulates Wnt signalling downstream of β-catenin stabilization. (**a**) TOPflash assay using whole embryos (stage 10.5, 10 embryos were used for each sample). Four-cell stage embryos were animally injected with indicated reagents (150 pg TOPflash reporter; 50 pg pRL-TK; 40 ng Co MO; 40 ng Uch37 MO; 20 pg Wnt8 mRNA; 1 ng Re.Uch37 mRNA). (**b**) Expression levels of Wnt target genes (siamois and nodal3.1) were examined by RT-PCR analysis using *Xenopus* animal cap tissues. Two-cell stage embryos were animally injected with indicated reagents (5 pg Wnt8 mRNA; 20 ng Co MO; 20 ng Uch37 MO; 1 ng Re.Uch37 mRNA). Animal cap explants were isolated at stage 9 and cultured until stage 11. WE, Whole embryos; (-), -RT; ODC, ornithine decarboxylase loading control. Full images are presented in Supplementary information (Fig. S12) (**c**) Axis duplication assay. Four-cell stage embryos were injected in one ventrovegetal blastomere with indicated reagents (5 pg Wnt8 mRNA; 1 ng Uch37 mRNA; 20 ng Uch37 MO). See also Table S2. (**d**) Quantified result of c. (**e**) TOPflash assay in HepG2 cells. After transfection of reporter constructs into stable cells (sh Control or sh Uch37) for 48 h, luciferase activity was measured. Knockdown of β-catenin indicates a positive control for downregulation of Wnt activity. (**f**) Quantitative real-time PCR (qPCR) analysis for the expression of Wnt-target genes (cyclinD1 and c-myc) in stable HepG2 cells. Knockdown of β-catenin indicates a positive control for downregulation of Wnt activity. The quantities of indicated mRNA were normalized by β-actin. Data represent average values from three independent experiments performed. Error bars indicate standard deviations of triplicate. *p < 0.002; **p < 0.001 (two-tailed Student’s ttest). (**g**) Axis duplication assay. Four-cell stage embryos were injected in one ventrovegetal blastomere with indicated reagents (5 pg Wnt8 mRNA; 200 pg Dvl2 mRNA; 25 pg β-catenin mRNA; 20 ng Co MO; 20 ng Uch37 MO). See also Supplementary Fig. S5a and Table S3).

Deubiquitinating enzymes (DUBs) are known to be involved in various signalling pathways by regulating the ubiquitination status of signal transducers^19^,^20^. Therefore, we hypothesized that defects observed in Uch37 morphants were due to attenuated deubiquitnation of Uch37 target proteins and subsequent failure of signal transduction. Before testing this hypothesis, we sought to find the signalling pathway that is specifically regulated by Uch37 using clues from the mesodermal defects of Uch37 morphants. Wnt8, BMP4, Xnr1 and eFGF signals are known to be involved in mesoderm development. The former two signals are required for dorso-ventral patterning^21^,^22^,^23^,^24^, whereas the latter two signals are important for mesoderm induction^25^. We, therefore, examined knockdown effects of Uch37 on each signalling pathway through RT-PCR analyses using *Xenopus* animal explants. Notably, we found that Uch37 MO antagonized expression of the Wnt target gene siamois but did not affect expression of target genes regulated by other signals (Supplementary Fig. S4a–d), suggesting that endogenous Uch37 is specifically required for Wnt8 signalling. These results were further validated by Wnt reporter assays using a TOPflash reporter containing TCF binding sites (Fig. 1a) and RT-PCR analysis (Fig. 1b) in *Xenopus*. Wnt8-mediated induction of reporter activity (Fig. 1a) and expression of Wnt target genes siamois and nodal3.1 (also known as Xnr3) (Fig. 1b) were drastically reduced by Uch37 MO, whereas addition of Re.Uch37 mRNA restored inhibitory effects of Uch37 MO (Fig. 1a,b). In addition, we monitored and quantified the effect of overexpression or knockdown of Uch37 on the formation of a secondary axis induced by Wnt8 in the ventral region. Ectopic expression of Uch37 mRNA enhanced the ability of Wnt8 to form a secondary axis, whereas depletion of Uch37 by Uch37 MO efficiently inhibited it (Fig. 1c,d, Table S2).

The influence of Uch37 on Wnt signalling was further assessed in HepG2 liver cancer cells. HepG2 cells are known to have hyperactivated Wnt-signalling^26^. Consistent with our *in vivo* analyses, we found that stable knockdown of Uch37 in HepG2 cells using short hairpin RNAs targeting Uch37 (shUch37; Supplementary Fig. S4e) led to a decrease in Wnt reporter activity and subsequent expression of Wnt target genes including cyclinD1 and c-myc (Fig. 1e,f). We also observed that downregulation of Uch37 inhibited ectopic Wnt activity that was induced by constitutively active LRP6 (LRP6ΔN) and the GSK3β inhibitor LiCl (Supplementary Fig. S4f,g). Together, these *in vivo* and *in vitro* results suggest that Uch37 is a novel positive regulator of Wnt signalling.

Next, we tried to elucidate where Uch37 resides within the Wnt signalling cascade. For that purpose, we analysed the effect of Uch37 knockdown on forced Wnt signalling mediated by Wnt8, Dishevelled2 (Dvl2) or β-catenin mRNAs (Fig. 1g, Supplementary Fig. S5a–c, Table S3). As a result, Uch37 knockdown successfully blocked both secondary axis formation and activation of Wnt target genes by Wnt8, Dishevelled2 (Dvl2) or β-catenin, strongly suggesting that Uch37 functions downstream of the β-catenin destruction complex. To further validate this hypothesis, we tested whether Uch37 affects the stabilization of β-catenin protein by performing overexpression or knockdown experiments using Uch37 mRNA or its MO. Our results showed that levels of β-catenin protein were not altered by overexpression or knockdown of Uch37 (Supplementary Fig. S5d). Taken together, our results clearly demonstrate that Uch37 positively regulates Wnt signalling downstream of β-catenin stabilization.

### Uch37 specifically and directly interacts with Tcf7

**Figure 2:**
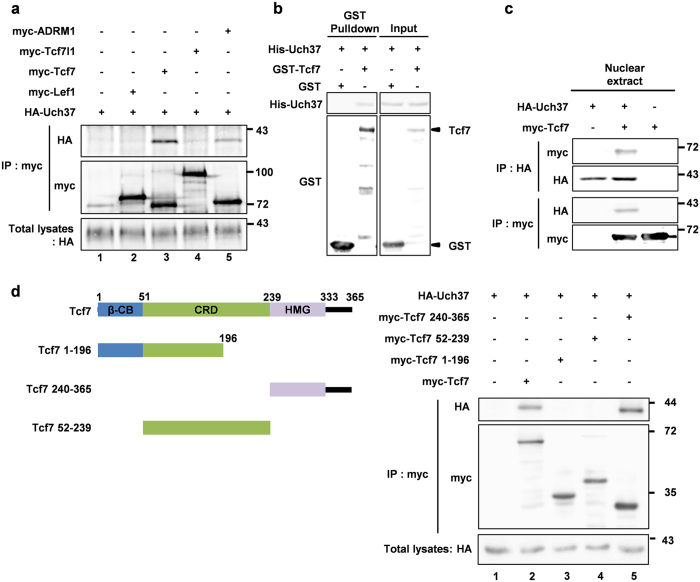
Uch37 physically interacts with Tcf7 in nucleus. (**a**) Co-IP assay in HEK293FT cells. Cells were transfected with indicated plasmids. myc-ADRM1 is known as an interacting partner of Uch37 and used as a positive control^11^. 48 h after transfection, cell lysates were precipitated with anti-myc antibody. (**b**) GST pulldown assay using purified proteins. His-Uch37 and GST-Tcf7 proteins were incubated and then subjected to immunoblotting. (**c**) Co-IP assay using nuclear extract of *Xenopus* gastrula embryo. One-cell stage embryos were injected with myc-Tcf7 (500 pg) and HA-Uch37 (500 pg) and cultured until stage 11, and then subjected to fractionation. Nuclear extracts were subjected to Co-IP assay using anti-myc or anti-HA antibody. (**d**) Co-IP assay using HEK293FT cells, HA-Uch37 and indicated myc-tagged truncated mutants of Tcf7 were transfected. Cell lysates were immunoprecipitated with anti-myc antibody. Truncated mutants of Tcf7 are depicted on the left. Tcf7, a full length; Tcf7 1–196, amino acids 1–196; Tcf7 240–365, amino acids 240–365; Tcf7 52–239, amino acids 52–239. Full images of all Fig. 2 are presented in Supplementary information (Fig. S9).

Our results suggest that Uch37 is involved in the Wnt signalling pathway downstream of β-catenin. The target protein of Uch37, however, has not been identified. In order to address this concern, we tested the possibility that Uch37 interacts with a pool of Tcf/Lef proteins that are involved in both Wnt signalling downstream of β-catenin stabilization and mesoderm development during *Xenopus* gastrulation^10^. Co-immunoprecipitation (Co-IP) analysis in HEK293FT cells showed that Uch37 interacts with myc-Tcf7 (Fig. 2a lane 3), but not with myc-Lef1 or myc-Tcf7l1 (Fig. 2a lane 2, 4). Moreover, GST-pull down assay using purified recombinant Uch37 (His-Uch37) and Tcf7 (GST-Tcf7) proteins demonstrated a direct interaction between Uch37 and Tcf7 (Fig. 2b). We further investigated Uch37 and Tcf7 subcellular localization in *Xenopus* embryo. Fractionation and immunostaining analyses in *Xenopus* gastrula showed that Uch37 co-localized with Tcf7 in the nucleus where Tcf7 protein exclusively resides despite both cytoplasmic and nuclear localization of Uch37 protein (Supplementary Fig. S6a and S6b), raising the possibility that the nuclear pool of Uch37 regulates Tcf7 to activate Wnt signalling. Moreover, Co-IP analysis using nuclear extracts of gastrula embryos provided additional evidence that these proteins bind to each other in the nucleus (Figs 2c and S6a).

We next determined the region of Tcf7 responsible for the interaction with Uch37. Tcf/Lef proteins possess four distinct domains including a β-catenin binding domain, context-dependent regulatory domain (CRD), high-mobility group (HMG) DNA-binding domain and a C-terminal extension^9^,^27^. Co-IP analysis using various deletion mutant forms of Tcf7 showed that a Tcf7 mutant form containing the HMG and C-terminal extension domains (Tcf7 240–365) bound to Uch37 with the same affinity as full-length Tcf7 (Fig. 2d lane 2, 5). However, neither the N-terminal half of Tcf7 protein (Tcf7 1–196; Fig. 2d lane 3), nor a CRD fragment of Tcf7 protein (Tcf7 52–239; Fig. 2d lane 4) interacted with Uch37. Co-IP analysis with other Tcf7 fragments further revealed that either partial deletion of C-terminal extension (Δ334–351) or HMG (Tcf7 325–365) lost the ability to interact with Uch37 (Supplementary Fig. 6c). These results also support the specific interaction of Uch37 with Tcf7, but not with other Tcfs including Lef1 and Tcf7l1, because alternative splicing at the C-terminus in Tcf/Lef gives rise to isoforms that have a variety of C-termini^9^. Taken together, our results suggest that Uch37 is a novel direct binding partner of Tcf7 protein and demonstrate that HMG domain and C-terminal extension (amino acids 240–365) of Tcf7 are important for this interaction.

### Uch37 regulates polyubiquitination of Tcf7 without affecting protein stability

**Figure 3:**
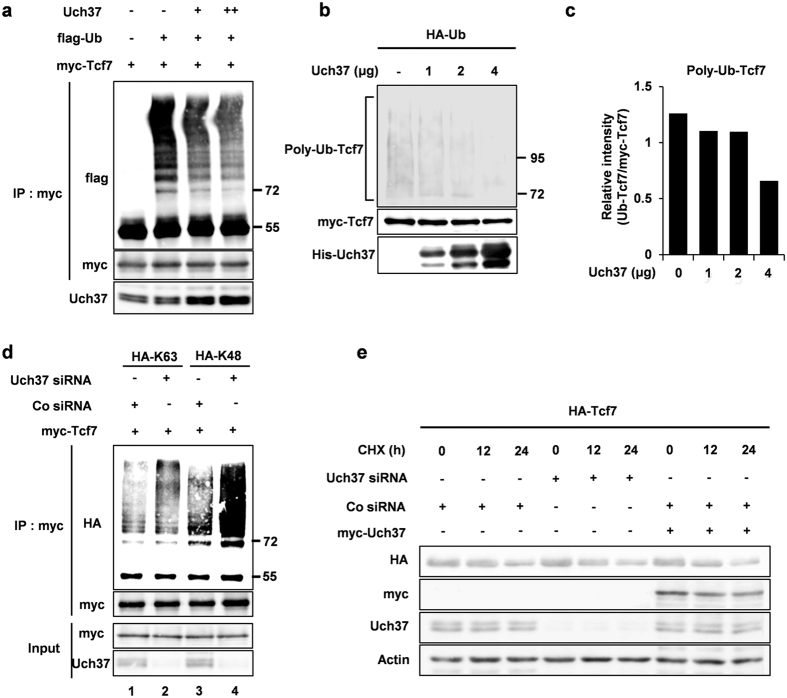
Uch37 deubiquitinates Tcf7 protein, but is not involved in protein stabilization. (**a**) *In vivo* ubiquitination assay in HEK293FT cells. Cells were transfected with indicated plasmids (1 μg myc-Tcf7; 3 μg flag-Ub; 2 μg and 4 μg Uch37). After 48 h, samples were prepared as described in materials and methods and then precipitated with anti-myc antibody. (**b**) *In vitro* ubiquitination assay. HEK293FT cells were co-transfected with both myc-Tcf7 and HA-Ub plasmids to express polyubiquitinated Tcf7. After 48 h, cell lysates were immunoprecipitated using anti-myc antibody to prepare Polyubiquitinated Tcf7. Precipitated polyubiqutinated Tcf7 was then incubated with indicated amount of His-Uch37 protein. (**c**) Signal intensity of polyubiquitinated Tcf7 in b was quantified using image J software. (**d**) *In vivo* ubiquitination assay in HEK293FT cells. Cells were transfected with indicated plasmids and siRNAs. After 72 h, total proteins were precipitated with anti-myc antibody. Cell lysis and detailed procedures are described in materials and methods. In HA-K63, all lysines except Lys-63 were mutated to arginines. In HA-K48, all lysines except Lys-48 were mutated to arginines. (**e**) Pulse-chase test using HEK293FT cells. 48 hours after the transfection as indicated, 100 μg/ml cycloheximide (CHX, sigma) was treated for 0, 12, and 24 h. myc-Tcf7, Uch37, and HA-Uch37 were monitored by western blot analysis, and Actin levels were used as a loading control. Full images of all Fig. 2 are presented in Supplementary information (Figs S10 and 11).

Having demonstrated that Uch37 is required for Wnt signalling and specifically interacts with Tcf7, we next hypothesized that Uch37 binds Tcf7 to serve as a bona fide DUB for Tcf7 protein during Wnt signalling. To examine this hypothesis, we performed *in vivo* ubiquitination assays using HEK293FT cells. Ubiquitination of Tcf7 protein is a prerequisite for the DUB function of Uch37. Our results clearly showed that Tcf7 is indeed highly polyubiquitinated when co-expressed with ubiquitin. Furthermore, increasing expression of Uch37 decreased the amount of polyubiquitin chains present on Tcf7 (Fig. 3a). To further examine if Uch37 directly deubiquitinates Tcf7, we conducted *in vitro* deubiquitination assays using immunopurified Tcf7-ubiquitin conjugates. Incubation of recombinant Uch37 protein with Tcf7-ubiquitin immunoprecipitates significantly reduced the high-molecular weight species of polyubiquitinated Tcf7 in a dose-dependent manner (Fig. 3b,c), suggesting that Uch37 removes polyubiquitin chains from Tcf7 directly.

It has been reported that polyubiquitination exerts two distinct functions on its target protein depending on the patterns of lysine linkages. The lysine 48 (K48)-linkage leads to degradation via the proteasome complex, whereas lysine 63 (K63)-linkage leads to proteasome-independent regulation without affecting stability^28^. Intriguingly, previous studies have shown that Uch37 removes either K48-linked or K63-linked polyubiquitin chains from target proteins to mediate proteolysis or non-proteolytic regulation, respectively^13^,^17^,^18^. We, therefore, wanted to determine the type of the ubiquitin linkage targeted on Tcf7 by Uch37. For this purpose, we conducted *in vivo* ubiquitination assays with two mutant forms of ubiquitin that only mediate either K63-linkage or K48-linkage^29^. Interestingly, we found that depletion of endogenous Uch37 promoted both forms of polyubiquitination linkages of Tcf7, compared with control cells (Fig. 3d lane 2, 4). Consistent with this result, overexpression of Uch37 eliminated both K63- and K48-linked polyubiquitin chains from Tcf7protein (Fig. S7a). Because our results suggested the possibility that Uch37 enhances resistance of Tcf7 to proteasomal degradation by removing K48- linked polyubiquitin chains from Tcf7, despite its removal activity on K63- linked polyubiquitin chains, we next examined if Uch37 stabilizes Tcf7 protein. Intriguingly, neither overexpression nor knockdown of Uch37 affected steady-state levels of Tcf7 protein in HEK293FT cells (Fig. 3e). Consistent with these results, we found that increasing amounts of Uch37 did not affect the level of Tcf7 protein in *Xenopus* cap explants (Fig. S7b). Together, these results support our hypothesis that Uch37 is a bona fide DUB of Tcf7 protein by detaching both K48- and K63- linkages from Tcf7. However, Uch37 activity does not appear to affect Tcf7 protein stability.

### Deubiquitinating activity of Uch37 is required for DNA binding of Tcf7

**Figure 4:**
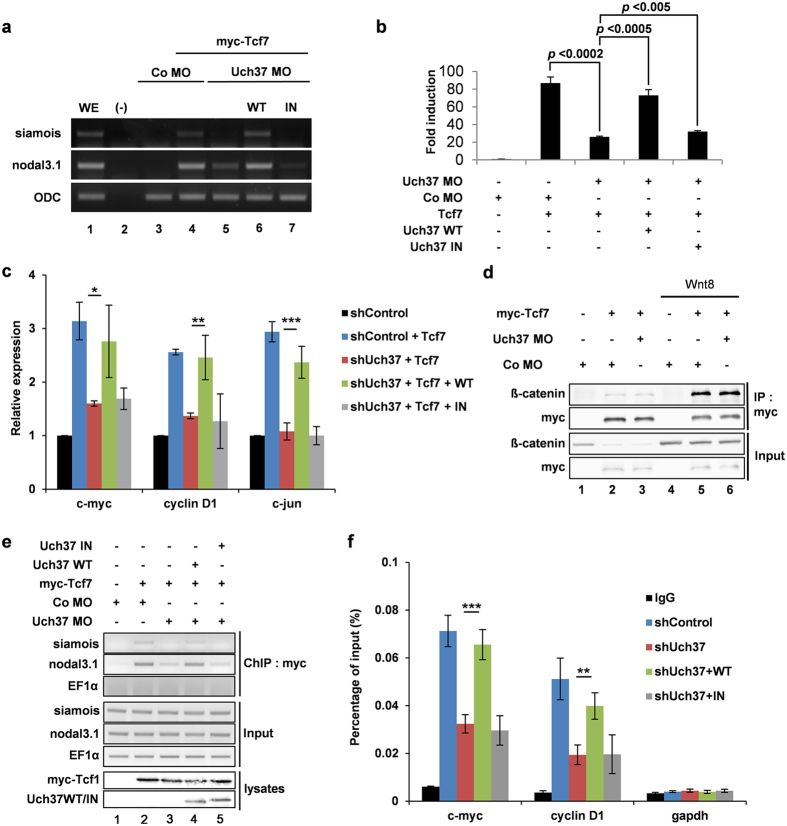
Deubiquitinating activity of Uch37 is required for Tcf7-mediated gene transcription by mediating DNA-binding of Tcf7. (**a**) Expression levels of Wnt target genes (siamois and nodal3.1) were examined by RT-PCR analysis using *Xenopus* animal cap tissues. Embryos were animally injected at four-cell stage. Animal caps were isolated at stage 9, grown to stage 11. Injected reagents are as follows, 25 pg myc-Tcf7 mRNA; 20 ng Co MO; 20 ng Uch37 MO; 1 ng wild type Uch37 mRNA (WT); 1 ng catalytically inactive Uch37 mRNA (IN). Full images are presented in Supplementary information (Fig. S12). (**b**) TOPflash assay using whole embryos (stage 10.5, 10 embryos). Embryos were animally injected at four-cell stage. 150 pg TOPflash reporter; 50 pg pRL-TK; 40 ng Co MO; 40 ng Uch37 MO; 50 pg Tcf7 mRNA; 1 ng wild type of Uch37 mRNA (WT); 1 ng catalytically inactive Uch37 mRNA (IN). (**c**) qPCR analysis for the expression of Wnt-target genes (c-myc, cyclinD1 and c-jun) in stable HepG2 cells. Tcf7 was transiently transfected alone or co-transfected with wild type of Uch37 or catalytically inactive Uch37. Error bars indicate standard deviations of triplicate. *p < 0.05; **p < 0.02; ***p < 0.003 (two-tailed Student’s t test). (**d**) Co-IP assay using *Xenopus* embryos (stage 11). Two-cell stage embryos were animally injected with indicated reagents (1 ng myc-Tcf7 mRNA; 20 pg Wnt8 mRNA; 40 ng Co MO; 40 ng Uch37 MO). Total proteins were precipitated with anti-myc antibody. Active state of Wnt signalling is indicated with enhanced β-catenin level in input panel. Full images are presented in Supplementary information (Fig. S11). (**e**) ChIP assay using *Xenopus* embryos (stage 11). 70 embryos were injected at two-cell stage as indicated (25 pg myc-Tcf7 mRNA; 1 ng wild type of Uch37 (WT); 1 ng catalytically inactive Uch37 (IN); 40 ng Co MO; 40 ng Uch37 MO). Lysates were precipitated with anti-myc antibody. Precipitated Wnt target DNAs were analysed by PCR. EF1α was used as a control for specificity. Full images are presented in Supplementary information (Fig. S11). (**f**) ChIP assay using stable HepG2 cells. Cells were transfected with either wild type of Uch37 (WT) or catalytically inactive Uch37 (IN). Lysates were precipitated with normal rabbit IgG or anti-Tcf7 antibody. DNA-binding of Tcf7 was assessed by qPCR. gapdh was used as a negative control. **p < 0.02; ***p < 0.003 (two-tailed Student’s t test).

Because Uch37 mediates deubiquitination of Tcf7 protein without affecting its stability, we next sought to elucidate the non-proteolytic role of Uch37-mediated deubiquitination of Tcf7 and subsequent activation of Wnt signalling. We hypothesized that deubiquitinating activity of Uch37 is involved in the ability of Tcf7 to activate transcription of Wnt target genes. To test this hypothesis, we first depleted endogenous Uch37 to suppress Tcf7-mediated activation of target genes (Fig. 4a) and Tcf7-mediated reporter activity (Fig. 4b). Next, either wild type Uch37 (WT) or catalytically inactive Uch37 (IN)^30^ was reintroduced to determine if Uch37 could rescue Wnt signalling. RT-PCR analysis using *Xenopus* tissues showed that only Uch37 WT mRNA, but not IN mRNA, efficiently rescued Uch37 MO-mediated reduction of siamois and nodal3.1 expression (Fig. 4a lane 6, 7). Moreover, Wnt reporter activity in *Xenopus* embryos was completely rescued by Uch37 WT, but not by Uch37 IN mRNA (Fig. 4b). We also confirmed these results in HepG2 cells with stable knock-down of endogenous Uch37. Reintroduction of Uch37 WT restored expression of Tcf target genes including c-myc, cyclinD1 and c-jun, whereas Uch37 IN did not (Fig. 4c). These results indicate that the deubiquitinating activity of Uch37 is required for Tcf7 mediated transcriptional activation of Wnt target genes.

To further elucidate the detailed regulatory mechanism by which Uch37 regulates the transcriptional activity of Tcf7, we analysed the influence of Uch37 on the formation of Tcf7-β-catenin complexes that mediate transcription of target genes in the Wnt signal cascade^10^,^27^. Co-IP analysis using *Xenopus* embryos showed that depletion of Uch37 did not affect binding affinity between endogenous β-catenin and myc-Tcf7 under both Wnt-inactive and Wnt-activated condition (Fig. 4d lane 3, 6), implying that Uch37 function is dispensable for the interaction between Tcf7 and β-catenin. We then investigated if Uch37 affects chromatin occupancy of Tcf7 to the Wnt target gene promoter, because ubiquitination of transcription factors often serves as a signalling cue for stripping transcription factors from DNA^31^,^32^,^33^. To address this concept, we performed chromatin-IP (ChIP) analysis using *Xenopus* embryos. siamois and nodal3.1 were chosen to monitor chromatin occupancy of Tcf7 due to the presence of Tcf/Lef binding sites in the promoter region^34^,^35^. Importantly, we observed that knockdown of Uch37 by Uch37 MO inhibited binding of Tcf7 to target gene promoters (Fig. 4e lane 3). Reduced chromatin occupancy of Tcf7 by Uch37 MO was rescued by addition of Uch37 WT mRNA (Fig. 4e lane 4). However, equal concentrations of Uch37 IN mRNA did not rescue the MO effect (Fig. 4e lane 5). Similar results were also shown in HepG2 cells. Stable knockdown of endogenous Uch37 by shRNA (shUch37) interfered with the binding of endogenous Tcf7 to the promoter regions of c-myc and cyclin D1 in HepG2 cells (Fig. 4f). Disrupted DNA binding of Tcf7 was rescued by reintroduction of Uch37 WT, but not by Uch37 IN (Fig. 4f). Taken together, our data suggest that deubiquitinating activity of Uch37 is required for Tcf7-mediated transcriptional activation through regulation of Tcf7 chromatin occupancy.

### Uch37 regulates expression of ventrolateral mesoderm genes by regulating Wnt signalling in *Xenopus* gastrula

**Figure 5:**
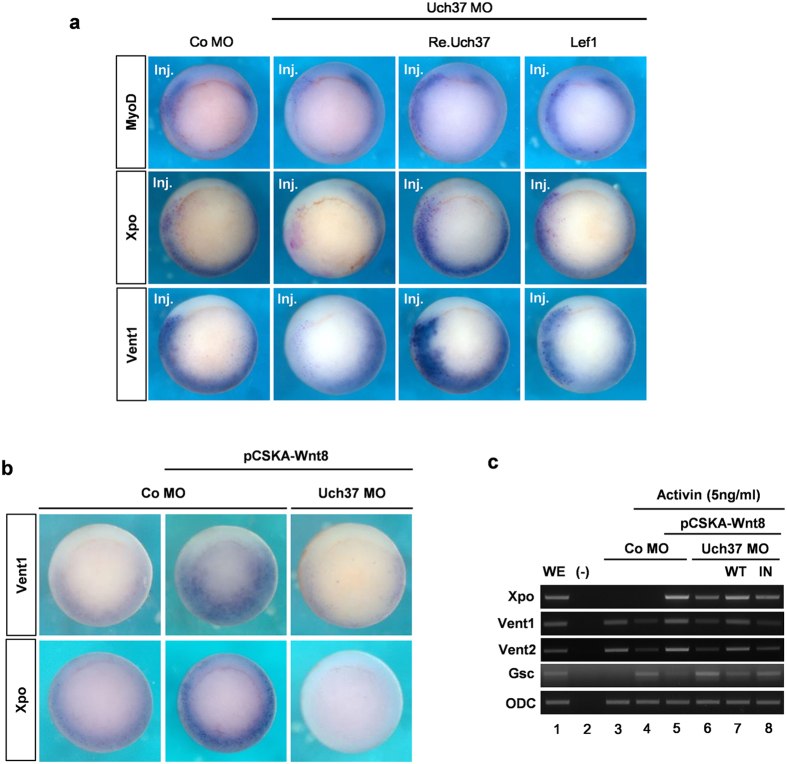
Uch37 is required for the expression of ventrolateral mesoderm genes by promoting zygotic Wnt signalling during *Xenopus* gastrulation. (**a**) Expressions of ventrolateral mesoderm genes including MyoD, Xpo, and Vent1. Vegetal-view of stage 10.5 embryos, MOs and mRNAs were unilaterally injected at four-cell stage embryos. β-galactosidase (LacZ) mRNA was co-injected to trace the injected site (Inj., injected site). (20 ng Co MO; 20 ng Uch37 MO; 100 pg Lef1; 2 ng Re.Uch37; 300 pg β-galactosidase). See also Table S4. (**b**) Expressions of Vent1 and *Xpo*. Vegetal-view of stage 10.5 embryo, Four-cell stage embryos were injected in VMZ and cultured until stage 10.5. Left panel, Co MO (40 ng); middle and right panels, injection of pCSKA-Wnt8 plasmids (500 pg) along with either Co MO (40 ng) or Uch37 MO (40 ng). See also Table S5. (**c**) RT-PCR analysis using animal cap explants. Four-cell stage embryos were animally injected with indicated reagents (20 ng Co MO; 20 ng Uch37 MO; 300 pg pCSKA-Wnt8; 1 ng Uch37 mRNA (WT); 1 ng a catalytically inactive Uch37 mRNA (IN)). Animal cap explants were dissected at stage 9, and then cultured in activin (5 ng/ml)–treated 1xMR until stage 11. *Gsc*, a dorsal mesoderm marker; Xpo, Vent1, and Vent2, ventrolateral mesoderm markers. Full images are presented in Supplementary information (Fig. S12).

The aim of our study was to identify the unknown function of Uch37 in *Xenopus* and loss-of-function assessment further revealed the requirement of Uch37 for mesoderm development. Therefore, we lastly analysed if this *in vivo* process requires Uch37 on account of our findings that suggest regulatory mechanisms of Uch37 for Wnt signalling. The best described function of Tcf7 in *Xenopus* mesoderm development is the regulation of expression of a subset of ventrolateral mesodermal marker genes including MyoD, Xpo and Vent1 during gastrulation^10^. We, therefore, analysed the effect of Uch37 knockdown on the expression of this subset of genes. Unilaterally injected Uch37 MO reduced the expression of MyoD, Xpo and Vent1. This reduction was rescued by co-injection of Re.Uch37 or Lef1 mRNA that does not interact with Uch37 (Fig. 5a, Table S4). Because both Tcf7 and Lef1 have functional redundancy for ventrolateral mesoderm development as a Wnt activator^10^, the rescue of MO effect by co-injection of Lef1 mRNA supports a specific role of Uch37 in Tcf7 regulation for ventrolateral mesoderm development. We also confirmed a consistent result that Uch37 MO specifically inhibited the formation of secondary axis induced by Tcf7 mRNA, but not by Lef1 mRNA (Supplementary Fig. S8a,b).

To further determine if this resulted from blockade of Wnt signalling, we used pCSKA-Wnt8 plasmids to induce excessive Wnt signalling after mid-blastula transition (MBT) when the ventrolateral mesoderm development is regulated^23^. Injection of pCSKA-Wnt8 strongly promoted expression of both Vent1 and Xpo^36^, whereas Uch37 MO drastically reduced them (Fig. 5b, Table S5). In addition, RT-PCR analysis using *Xenopus* tissues supported results of *in situ* hybridization analysis and further suggested that the reduction of ventrolateral mesoderm gene expression is due to the loss of Uch37-mediated transduction of Wnt signalling (Fig. 5c). Injection of pCSKA-Wnt8 transformed *Xenopus* ectoderm tissues into ventral mesoderm by inducing Xpo, Vent1 and Vent2 expression^25^,^37^ (Fig. 5c lane 5), and co-injection of Uch37 MO diminished the expression *of* Xpo *and* Vent1/2 (Fig. 5c lane 6). Importantly, reintroduction of Uch37 WT mRNA restored the ability of Wnt8 to induce Xpo, Vent1 and Vent2 expression, whereas Uch37 IN mRNA did not (Fig. 5c lane 7 and 8). We also examined the effect of Uch37 on chromatin occupancy of Tcf7 in the promoter of Vent2, which is a Tcf7 target gene in ventrolateral mesoderm^38^. As a result, Uch37 MO led to a decrease in DNA binding of Tcf7 and this reduced chromatin occupancy was rescued by Uch37 WT, but not by Uch37 IN mRNA (Supplementary Fig. S8c). Taken together, our results suggest that the deubiquitinating activity of Uch37 is required for Wnt8-mediated ventrolateral mesoderm development during *Xenopus* gastrulation.

## Discussion

Because the relationship between Wnt signalling and a myriad embryonic developmental processes in *Xenopus laevis* has been well demonstrated and established, *Xenopus laevis* has been considered the best *in vivo* system to easily and clearly identify gene functions involved in Wnt signalling^39^. In this study, we identified Uch37 as a novel positive regulator of Wnt signalling and revealed the detailed molecular mechanism regulating Wnt signalling in *Xenopus* embryos. Our Co-IP and GST pulldown analyses clearly demonstrated that Uch37 binds Tcf7 directly, and that the physical interaction requires the C-terminal region of Tcf7 protein that includes HMG domain and C-terminal extension. In respect to the involvement of this region in DNA-binding of Tcf protein, our observation strongly supports that Uch37 regulates DNA binding of Tcf7. In addition, the requirement of partial region in C-terminal extension (amino acids 334–351) of Tcf7 for the interaction may explain the binding specificity between Tcf7 and Uch37, which requires further studies to solidify this interpretation. Based on these observations, we identify Tcf7 as a novel binding partner of Uch37. Our study also shows that Uch37 is a bona fide DUB for Tcf7 by directly removing polyubiquitin chains from Tcf7. Intriguingly, *in vivo* ubiquitination assays using mutant ubiquitin constructs further showed that both K63- and K48-linked polyubiquitination of Tcf7 precedes Uch37 function and that Uch37 simultaneously detaches both polyubiquitin chains from Tcf7 protein. These results imply the dual function of Uch37 on both non-proteolytic and proteolytic regulation of Tcf7. However, gain- or loss-of function of Uch37 did not alter the steady-state levels of Tcf7 protein. We suggest two possible explanations for this unexpected finding. One possible explanation is that K63-linked ubiquitination counteracts the effects of K48-linked ubiquitination on Tcf7 for its accessibility to the proteasome complex. Previous reports suggest that polyubiquitin chains could interact with the proteasome with high affinity and mediate subsequent proteolysis^40^, and K63-linked polyubiquitin chains are less accessible compared to K48-linked polyubiquitin chains^41^. Therefore, it is possible that K63-linked polyubiquitination functions dominantly for non-proteolytic regulation of Tcf7 and may counteract the recruitment of Tcf7 to the proteasome by K48-linkage, subsequently maintaining steady-state levels of Tcf7 protein. Another explanation is the possible novel function of Uch37 on non-proteolytic regulation through K48-linked polyubiquitination. Although K48-linked polyubiquitin chains have been accepted as proteasome-dependent proteolytic signals in the classical view^40^, recent studies have also identified several unknown aspects of non-proteolytic functions for K48-linked polyubiquitin chains. For instance, it has been shown that transcriptional activity of two transcription factors, Met4 and Spt23, is regulated by a non-proteolytic function of K48-linked polyubiquitin chains^40^. Although we could not clearly identify this intriguing possible mechanism of Uch37 in this study, we speculate that the identification of an E3 ligase that functions as a counter partner of Uch37 could allow elucidation of Uch37-mediated detailed regulatory mechanism of Tcf7 to fully answer this question. Nonetheless, our study demonstrates that the non-proteolytic function of Uch37 is crucial for transcriptional activation of Tcf7 protein.

Previous studies have shown that chromatin occupancy of Tcf/Lef family members is regulated by either interaction with β-catenin^42^,^43^ or phosphorylation^38^,^44^,^45^,^46^. Tcf7l1 forms a complex with β-catenin and thereby loses chromatin occupancy in mouse embryonic stem cells and breast cancer cells^42^, whereas recombinant β-catenin strongly promotes binding of Lef1 to chromatin templates *in vitro*^43^. Meanwhile, a negative regulatory role of phosphorylation on DNA-binding by Tcf/Lef proteins has been reported in frog and human cultured cells. Phosphorylation of Lef1, Tcf7l1 and Tcf7l2 (formerly named Tcf4) proteins on their consensus residues (S/TP-) by NLK and HIPK2 kinases disturbs their binding to DNA^38^,^44^,^45^,^46^. However, β-catenin binding, phosphorylation, or other unknown mechanisms that regulate DNA binding by Tcf7 have not been studied. In support of this, our study suggests that Tcf7 achieves chromatin occupancy, not only through β-catenin interaction or phosphorylation, but also through deubiquitination by Uch37. Our findings suggest an important new aspect of non-proteolytic ubiquitination that manipulates chromatin occupancy of Tcf7 and further provides a potential involvement of ubiquitination for regulating other Tcf/Lef family members.

Although a majority of the results in this study focused on the developing *Xenopus* embryo, we also revealed that Uch37 is required for the regulation of Wnt signalling in HepG2, a liver carcinoma cell. Importantly, previous clinicopathologic and *in vitro* approaches have shown that Uch37 is overexpressed in hepatocellular carcinoma (HCC) type of cancer tissues and cells, thus Uch37 has been suggested as a marker gene for HCCs^47^,^48^. However, despite other previous findings in HCCs that aberrant regulation of Wnt signalling influences oncogenesis^26^,^49^,^50^, the link between Uch37 and Wnt signalling in cancer has not been demonstrated. Our data gives clear evidence that excessive Wnt signalling in HepG2 cells^26^,^49^ is relieved by suppression of Uch37 and the efficacy of Uch37 comes from inhibition of endogenous Tcf7 binding to the promoter of Wnt target genes. These results raise the strong possibility that abnormal Uch37 function may be critical for Wnt-mediated oncogenesis of HCCs.

Based on our findings detailing a molecular mechanism of Uch37, we further verified these under physiological conditions. Zygotic activation of Wnt8 signalling is required for the ventrolateral mesoderm development from late blastula and gastrula stages of *Xenopus* embryo^21^,^51^. Knockdown of Wnt components, introduction of dominant-negative Wnt components and treatment of LiCl, a GSK3β inhibitor, consistently support the notion that Wnt signalling is required for expression of a subset of ventrolateral mesoderm genes^6^,^7^,^10^,^21^,^52^. Consistent with these previous reports, our results also revealed that knockdown of Uch37 blocked the expression of ventrolateral mesoderm genes, confirming the role of Uch37 as a novel Wnt component. Moreover, through comparative studies using wild type Uch37 and catalytically inactive Uch37, we further demonstrate that the deubiquitinating activity of Uch37 is essential for the induction of Wnt8-mediated ventrolateral mesoderm genes. Together, these results demonstrate defective phenotypes caused by ventrally depleted Uch37. Intriguingly, although knockdown of Uch37 at the dorsal region did not affect blastopore closure and expression of dorsal mesoderm genes during gastrulation, we found that these morphants have a tendency to show neural tube closure defects and subsequent malformation of anterior structures (data not shown). We carefully interpret these results as another developmental function of Uch37 at the onset of neurulation.

However, our findings regarding the physiological role of Uch37 during gastrulation coincide with previous reports showing that neither suppression of Wnt8 by a dominant negative form of mRNA nor excessive activation of Wnt8 by pCSKA-Wnt8 affects the expression of dorsal mesoderm genes during *Xenopus* gastrulation^21^. In addition, we confirmed the rescue activity of ectopically expressed Lef1 on the expression of ventrolateral mesoderm genes against Uch37 MO and observed resistance to inhibition of Wnt activity by Uch37 depletion. These findings strongly imply the specific requirement of Uch37 for Tcf7 regulation.

**Figure 6:**
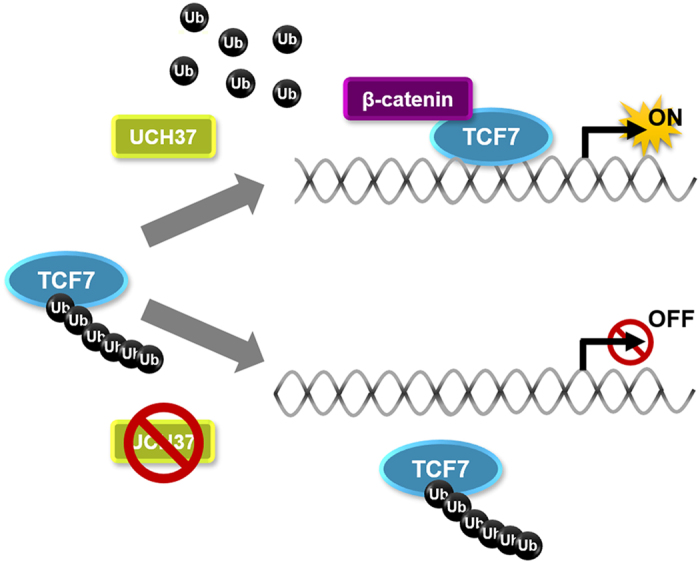
Proposed model depicting how Uch37 regulates Tcf7 protein. Uch37 interacts with Tcf7 and removes polyubiquitin chain from Tcf7 protein. As a result, Tcf7 stably binds target DNA for gene transcription. However, absence of Uch37 promotes polyubiquitination on Tcf7 protein, causing transcriptional silence.

In conclusion, our study uncovers a pivotal role of Uch37 in Wnt signal transduction and Wnt-related developmental processes. Uch37 is required for DNA binding of Tcf7 by mediating deubiquitination of Tcf7. As depicted in Fig. 6, Tcf7 might be polyubiquitinated by an unknown E3 ligase, which represses DNA-binding of Tcf7 upstream of Uch37 function. This repression could be relieved by Uch37 through removing polyubiquitin chains and allowing Tcf7 to fully activate Wnt signalling. *In vivo* analyses further demonstrated that an endogenous function of Uch37 is involved in ventrolateral mesoderm development mediated by zygotic Wnt8 signalling. To better understand physiological functions of Tcf7 ubiquitination and the significance in Wnt signalling regulation, future studies could identify an E3 ligase that may act as a counter-partner of Uch37 and the target residue. Moreover, because our study also gives new mechanistic insight into the oncogenic role of Uch37, further studies may elucidate the relationship between Uch37 and Wnt signalling in HCCs.

## Methods

### *In vitro* fertilization, microinjection, and cell culture

Eggs were obtained from *Xenopus laevis* (Nasco) primed with 800U of human chorionic gonadotropin (Sigma). *In vitro* fertilization was performed as described previously^53^, and developmental stages of the embryos were determined according to Nieuwkoop and Faber (1967). Microinjection was carried out in 0.33 Modified Ringer (MR) containing 4% Ficoll-400 (GE health care) using a PIL-100 Injector (HARVARD). Injected embryos were cultured in 0.33MR until stage 8 and then transferred to 0.1MR until they had reached the appropriate stage for the experiments.

HEK293FT and HepG2 cells were maintained in complete Dulbecco’s Modified Eagle’s medium (DMEM) supplemented with 10% fetal bovine serum (FBS, Hyclone), 100 units/ml penicillin, and 100 mg/ml streptomycin at 37 °C with 5% CO_2_. Cells were transfected using Lipofectamin 2000 (Invitrogen) and then subjected to each experiment after 48–72 h.

### Plasmids, mRNA, and knockdown materials

The complete coding regions of *Xenopus laevis* Uch37 (NM_001095597) and human Uch37 (NM_015984) were amplified by PCR and inserted into the pCS2+ (XhoI/XbaI), pCS2+ −6myc (XhoI/XbaI) or pCS4-3HA (BamH1/ClaI) vector. Catalytically inactive human Uch37 (IN) was previously described^30^ and generated by PCR-directed mutagenesis. To generate *Xenopus laevis* Tcf/Lef constructs, we amplified coding region of Lef1 (NM_001088655), Tcf7 (isoform C, AY163239), Tcf7l1 (NM_001087469) by PCR and then, inserted them into pCS2+ −6myc (Xho1/Xba1) vector. For *in vitro* assay (GST-pulldown assay and *In vitro* deubiquitination assay), we sub-cloned Uch37 and Tcf7 with pProEX HT (BamH1/Xho1) and pGEX-4T3 (EcoR1/Xho1) vectors respectably. For Capped mRNAs, constructs were digested with NotI, and then were synthesized *in vitro* using the mMessage mMachine Kit (Ambion). Antisense morpholino oligonucleotides (MOs) were purchased from Gene Tools. The Uch37 MO: 5′-TACCGCCCGTCACGTCCTCTTACCA-3′; the control MO: 5′-CCTCTTACCTCAGTTACAATTTATA-3′.

siRNA targeting Uch37 following 25‐nucleotide sequences were purchased from Invitrogen: sequences were purchased 5′-ACCGAGCTCATTAAAGGATTCGGTT-3′. The knockdown efficiency and specificity of Uch37 siRNA were confirmed previously^54^. siRNA targeting β-catenin following 21-nucleotide sequences were purchased from Bioneer: 5′-GGGTTCAGATGATATAAATTT-3′. The knockdown efficiency and specificity of β-catenin siRNA were confirmed previously^55^. Stable HepG2 cells expressing short hairpin RNA (shRNA) for Uch37 depletion were generated by infection of lentiviral package. shRNA sequence for Uch37 was: 5′-TCCCGACTTGACACGATATTT-3′. Information about shRNA sequence, cloning, viral packaging, and infection procedures are precisely described in GPP Web Potal (http://portals.broadinstitute.org/).

### Whole mount *in situ* hybridization and RT-PCR

Whole-mount *in situ* hybridization was performed according to a standard protocol^56^. Probes were labelled using digoxigenin-11-UTP and appropriate RNA polymerases. An antisense *in situ* probe against Uch37 was generated by linearizing the pBS‐KS-Uch37 construct with *Hin*dIII and transcribing using the T3 RNA polymerase.

For RT-PCR analyses, total RNA extraction and reverse transcription were performed as described previously^57^. PCR-conditions and primers are described in De Robertis’ homepage (http://www.hhmi.ucla.edu/derobertis/index.html). Uch37 primers: forward, 5′-TGCATCACGGTACATTAGGC-3′; reverse, 5′-CAGGAGGTCTGCAAATGTGA-3′.

For quantitative real time PCR using HepG2 cells, total RNA was isolated using TRIZOL reagent (Invitrogen) according to the manufacturer’s protocol. cDNAs were synthesized from total RNAs using M-MLV Reverse Transcriptase (Promega) with random primer (Promega). Quantitative real-time PCR was performed by using the StepOne real-time PCR system (Applied Biosystems), and relative quantity was analyzed by using comparative Ct methods described in the manufacturer’s manual. Information about PCR condition and primers used for Wnt target genes were described previously^58^.

### Immunoprecipitation, GST-pulldown assay, and preparation of *Xenopus* nuclear fraction

HEK293FT cells were transfected with the indicated constructs for 48 h and then resuspended using immunoprecipitation (IP) buffer, 2 mM Tris-Cl (pH7.5), 2 mM EDTA, 150 mM NaCl, 1% TritonX-100. Cells were disrupted and centrifuged in order to remove insoluble debris. The indicated antibodies were added to the supernatants and incubated at 4 °C for 6 h. Protein G-sepharose (Invitrogen) was added and the mixture was incubated at 4 °C overnight. The immunocomplexes bound to beads were washed four times with IP buffer. Mouse anti-myc monoclonal, Rabbit anti-HA polyclonal (Santa Cruz Biotechnology) were used for IP and Immunoblot analysis.

For purification of recombinant proteins, Uch37 (N-terminally histidine tagged) and Tcf7 (N-terminally GST-tagged) were respectively expressed in *Escherichia coli* (BL21) under 0.5 mM IPTG-treated condition at (His-Uch37, OD600 0.5 at 37 °C for 3 h in 250 ml LB; GST-Tcf7 OD600 0.5 at 18 °C for 12 h in 1 L LB). Cells were harvested by centrifugation and resuspended in lysis buffer (PBS pH 7.4, 5 mM β-ME, and 5% Glycerol). His-Uch37 was purified from the soluble fraction of the cell lysates using Ni-NTA agarose (Qiagen). Eluted protein was dialysed against 50 mM Tris (pH 7.4), 150 mM NaCl, 2 mM dithiothreitol (DTT) and 5% glycerol. For GST-Tcf7, cells were lysed by sonication in lysis buffer containing 1 mM PMSF. Cell lysates were cleared by centrifugation (18000 rpm, 1 h, 4 °C). Soluble fraction was incubated with 5 ml of Glutathione-Sepharose beads (Peptron) at 4 °C for 4 h. After that, beads were subjected to extensive washing with lysis buffer. GST-Tcf7 was eluted in PBS supplemented with 10 mM reduced glutathione and then, was dialysed against 50 mM Tris (pH 7.4), 150 mM NaCl, 2 mM DTT and 5% glycerol overnight at 4 °C. For GST-pulldown assay, approximately 3 μg of GST or GST-Tcf7 was incubated with His-Uch37 in IP buffer at 4 °C for 6 h. Glutathione-Sepharose beads (Peptron) were added to immobilize protein complexes. After incubation, beads were washed four times using IP buffer and then, samples were subjected to immunoblotting.

For preparation of *Xenopus* nuclear fraction, 70 embryos were lysed using cold fractionation buffer 10 mM HEPES (pH7.6), 3 mM MgCl_2_, 0.5% NP-40, 5% glycerol, 40 mM KCl, 2 mM DTT and then lysates were subjected centrifuge (3000 rpm, 5 min, 4 °C). After washing the pellet (nuclear fraction) 3 times with the fractionation buffer, the pellet was disrupted with IP buffer.

### *In vivo* and *In vitro* ubiquitination assay

A denaturing immunoprecipitation was performed by transfecting HEK293FT cells with Flag-Ub, HA-Ub K48 or HA-Ub K63 ubiquitin constructs that mediate poly-ubiquitination. After 48 h, cells were harvested with SDS lysis buffer 10 mM Tri-Cl (pH 7.5), 150 mM NaCl, 1% SDS, 1 mM DTT and then boiled at 100 °C for 5 min. Lysates were diluted with IP buffer to make 0.1% SDS as a final concentration, sonicated and finally centrifuged (at 14,000 rpm, for 20 min). 1 mg of supernatants was subjected to immunoprecipitation with indicated antibody at 4 °C for overnight. Then, Protein G sepharose (Invitrogen) was added and the mixture was incubated at 4 °C for 2 or 4 h. Beads were washed 4 times with IP buffer and boiled to be immunoblotted.

For *in vitro* ubiquitination analysis, both Tcf7 and HA-Ub were transfected to HEK293FT cells. After 48 h, cells were lysed with IP buffer. Cell lysates were clarified by centrifuge and then supernatants were sequentially incubated with indicated antibody and Protein G sepharose (Invitrogen) at 4 °C for 3 h. Beads were washed 3 times with *in vitro* DUB buffer (50 mM Tris-Cl (pH7.5), 150 mM NaCl, 1 mM EDTA and 1 mM DTT). Washed beads were incubated with indicated amount of purified Uch37 recombinant proteins at 37 °C for 2 h in 50 μl *in vitro* DUB buffer. The reaction was terminated by adding SDS sample buffer and subsequent boiling at 95 °C for 10 min. Samples were subjected to immunoblotting analysis.

### Chromatin immunoprecipitation (ChIP)

For ChIP assay using *Xenopus* embryos, detailed ChIP procedures including crosslinking condition, lysis buffer, and primers were based on previously published methods^38^,^59^,^60^. Two-cell stage embryos were injected with indicated mRNA or MO. 70 embryos (at stage 11) were sonicated, and then lysates were incubated with 2 μg of rabbit anti-myc antibody (Santa Cruz Biotechnology) at 4 °C overnight. For ChIP assay using HepG2 cells, we followed abcam protocol (X-ChIP protocol). 1 × 10^7^ cells were used for each ChIP reaction. Lysates were incubated with 4 μg of normal rabbit IgG (Santa Cruz Biotechnology) or Tcf7 (Cell signaling) at 4 °C overnight. Primers used were: c-myc, forward 5′-TCTCCCTGGGACTCTTGATCA-3′ and reverse 5′-TTTGACAAACCGCATCCTTGT-3′^26^; cyclinD1, forward 5′-CGGAATGAAACTTGCACAGG-3′ and reverse 5′-AGACGGCCAAAGAATCTCAG-3′^61^; gapdh, forward 5′-TAGGCCTTTGCCTGAGCAGTCCGGTGT-3′ and reverse 5′-TTGAGGCCTGAGCTACGTGCGCCCGTAA-3′^62^. PCR conditions were 95 °C (10 min) and 50 cycles of 95 °C (30 s) and 60 °C (1 min).

### Ethics Statement

The care of *Xenopus laevis* and manipulation of embryos were carried out according to standard protocols. All of the animal protocols were approved by the Pohang University of Science and Technology Institutional Animal Care and Use Committee (approval number: POSTECH-2016-0092).

## Additional Information

**How to cite this article**: Han, W. *et al*. Ubiquitin C-terminal hydrolase37 regulates Tcf7 DNA binding for the activation of Wnt signalling. *Sci. Rep.* **7**, 42590; doi: 10.1038/srep42590 (2017).

**Publisher's note:** Springer Nature remains neutral with regard to jurisdictional claims in published maps and institutional affiliations.

## Supplementary Material

Supplementary Information
