# Supplementary material for: Ubiquitin C-terminal hydrolase37 regulates Tcf7 DNA binding for the activation of Wnt signalling
Source: Sci Rep. 2017 Feb 15;7:42590. doi: 10.1038/srep42590 (PMC5309806; doi:10.1038/srep42590)
Supplement: Supplementary Information [file srep42590-s1.pdf]

Supplementary Information

12 Supplementary figures with legends and 5 Supplementary tables

Ubiquitin C-terminal hydrolase37 regulates Tcf7 DNA binding for the activation of Wnt signalling

Wonhee Han[1], Hyeyoon Lee[1] and Jin-Kwan Han[1*]

[1]Department of Life Sciences, Pohang University of Science and Technology, 77 Cheongam-Ro, Nam-Gu, Pohang, Gyeongbuk, 37673, Korea

[*] Author for correspondence (jkh@postech.ac.kr)

Figure S1

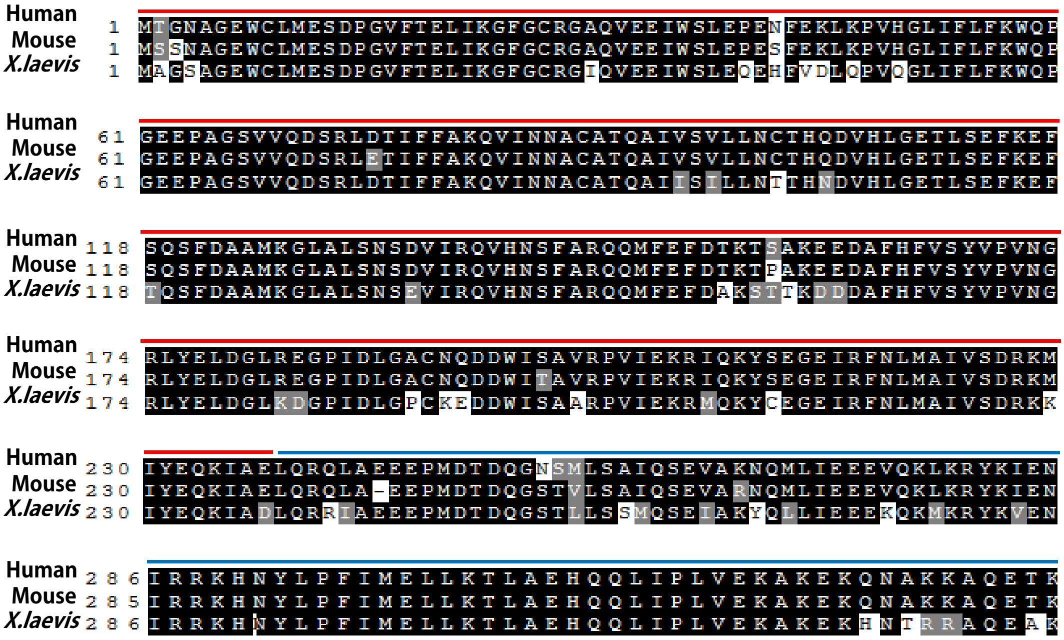
Human 1 MTGNAGEWCLMESDPGVFTELIKGFGCRGAQVEEIWSLEPENFEKLKPVHGLIFLFKWQP
Mouse 1 MSSNAGEWCLMESDPGVFTELIKGFGCRGAQVEEIWSLEPESFEKLKPVHGLIFLFKWQP
X.laevis 1 MAGSAGEWCLMESDPGVFTELIKGFGCRGIQVEEIWSLEQEHFVDLQPVQGLIFLFKWQP
Human 61 GEEPAGSVVQDSRLDTIFFAKQVINNACATQAIVSVLLNCTHQDVHLGETLSEFKEF
Mouse 61 GEEPAGSVVQDSRLETIFFAKQVINNACATQAIVSVLLNCTHQDVHLGETLSEFKEF
X.laevis 61 GEEPAGSVVQDSRLDTIFFAKQVINNACATQAIISILLNTTHNDVHLGETLSEFKEF
Human 118 SQSFDAAMKGLALSNSDVIRQVHNSFARQQMFEFDTKTSAKEEDAFHFVSYVPVNG
Mouse 118 SQSFDAAMKGLALSNSDVIRQVHNSFARQQMFEFDTKTPAKEEDAFHFVSYVPVNG
X.laevis 118 TQSFDAAMKGLALSNSEVIRQVHNSFARQQMFEFDAKSTTKDDDAFHFVSYVPVNG
Human 174 RLYELDGLREGPIDLGACNQDDWISAVRPVIEKRIQKYSEGEIRFNLMAIVSDRKM
Mouse 174 RLYELDGLREGPIDLGACNQDDWITAVRPVIEKRIQKYSEGEIRFNLMAIVSDRKM
X.laevis 174 RLYELDGLKDGPIDLGPCKEDDWISAARPVIEKRMQKYCEGEIRFNLMAIVSDRKK
Human 230 IYEQKIAELQRQLAEEEPMDTDQGNSMLSAIQSEVAKNQMLIEEEVQKLKRYKIEN
Mouse 230 IYEQKIAELQRQLA-EEPMDTDQGSTVLSAIQSEVARNQMLIEEEVQKLKRYKIEN
X.laevis 230 IYEQKIADLQRRIAEEEPMDTDQGSTLLSSMQSEIAKYQLLIEEEKQKMKRYKVEN
Human 286 IRRKHNYLPFIMELLKTLAEHQQLIPLVEKAKEKQNAKKAQETK
Mouse 285 IRRKHNYLPFIMELLKTLAEHQQLIPLVEKAKEKQNAKKAQETK
X.laevis 286 IRRKHNYLPFIMELLKTLAEHQQLIPLVEKAKEKHNTRRAQEAK

Figure S2

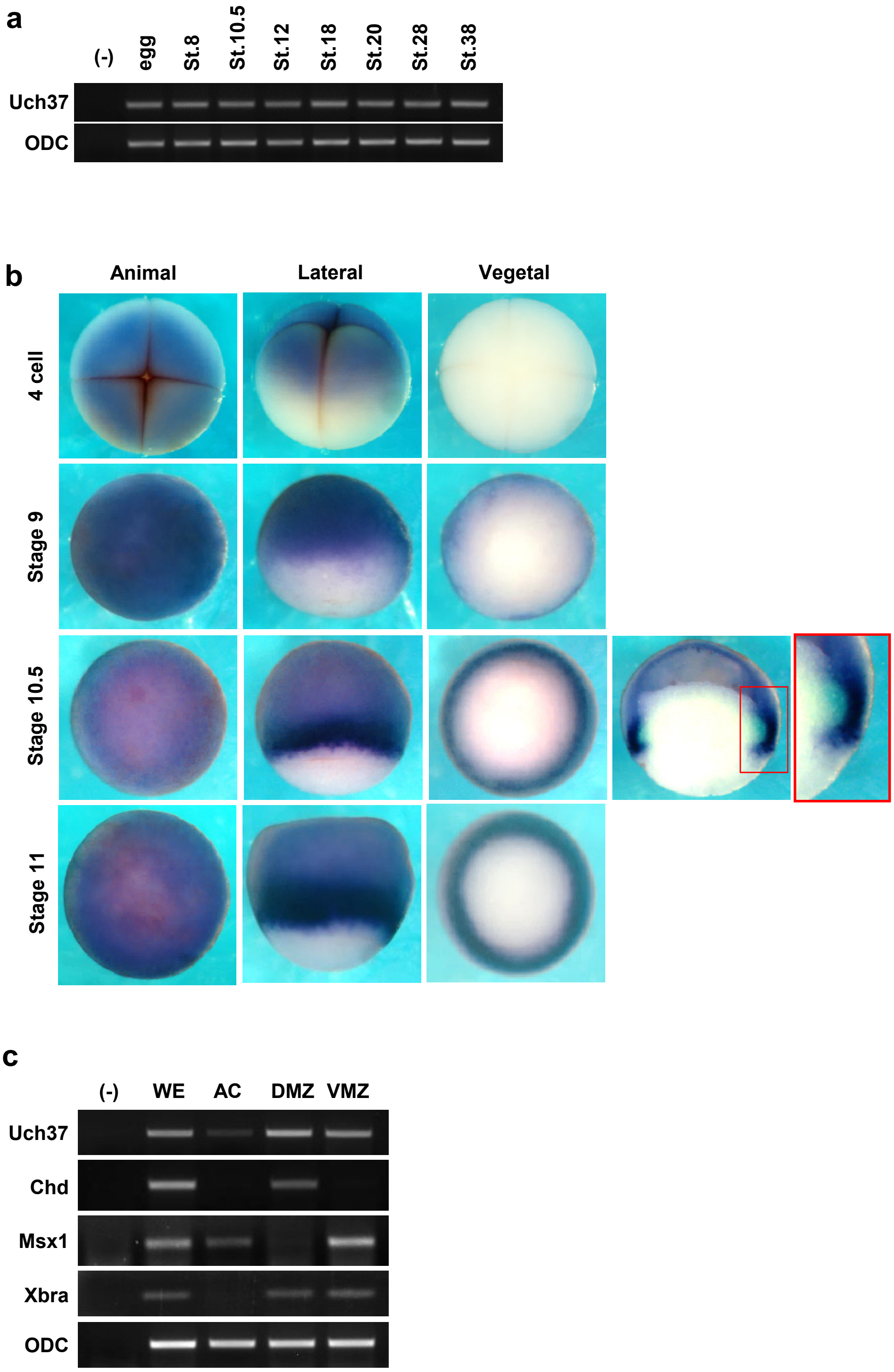
a
(-)
egg
St.8
St.10.5
St.12
St.18
St.20
St.28
St.38
Uch37
ODC
b
Animal
Lateral
Vegetal
4 cell
Stage 9
Stage 10.5
Stage 11
c
(-)
WE
AC
DMZ
VMZ
Uch37
Chd
Msx1
Xbra
ODC

Figure S3

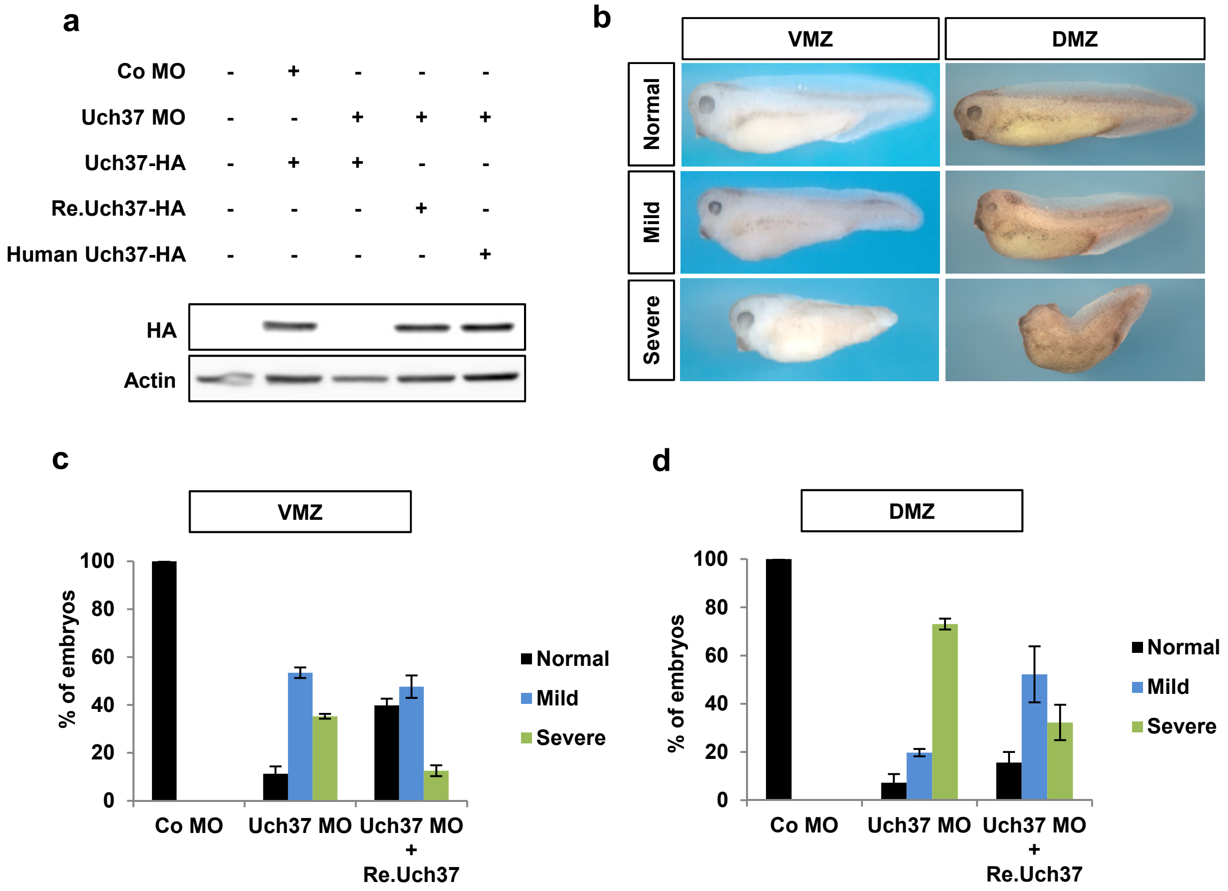

a
Co MO - + - - -
Uch37 MO - - + + +
Uch37-HA - + + - -
Re.Uch37-HA - - - + -
Human Uch37-HA - - - - +
HA
Actin
b
VMZ
DMZ
Normal
Mild
Severe
c
VMZ
% of embryos
100
80
60
40
20
0
Co MO
Uch37 MO
Uch37 MO + Re.Uch37
Normal
Mild
Severe
d
DMZ
% of embryos
100
80
60
40
20
0
Co MO
Uch37 MO
Uch37 MO + Re.Uch37
Normal
Mild
Severe

Figure S4

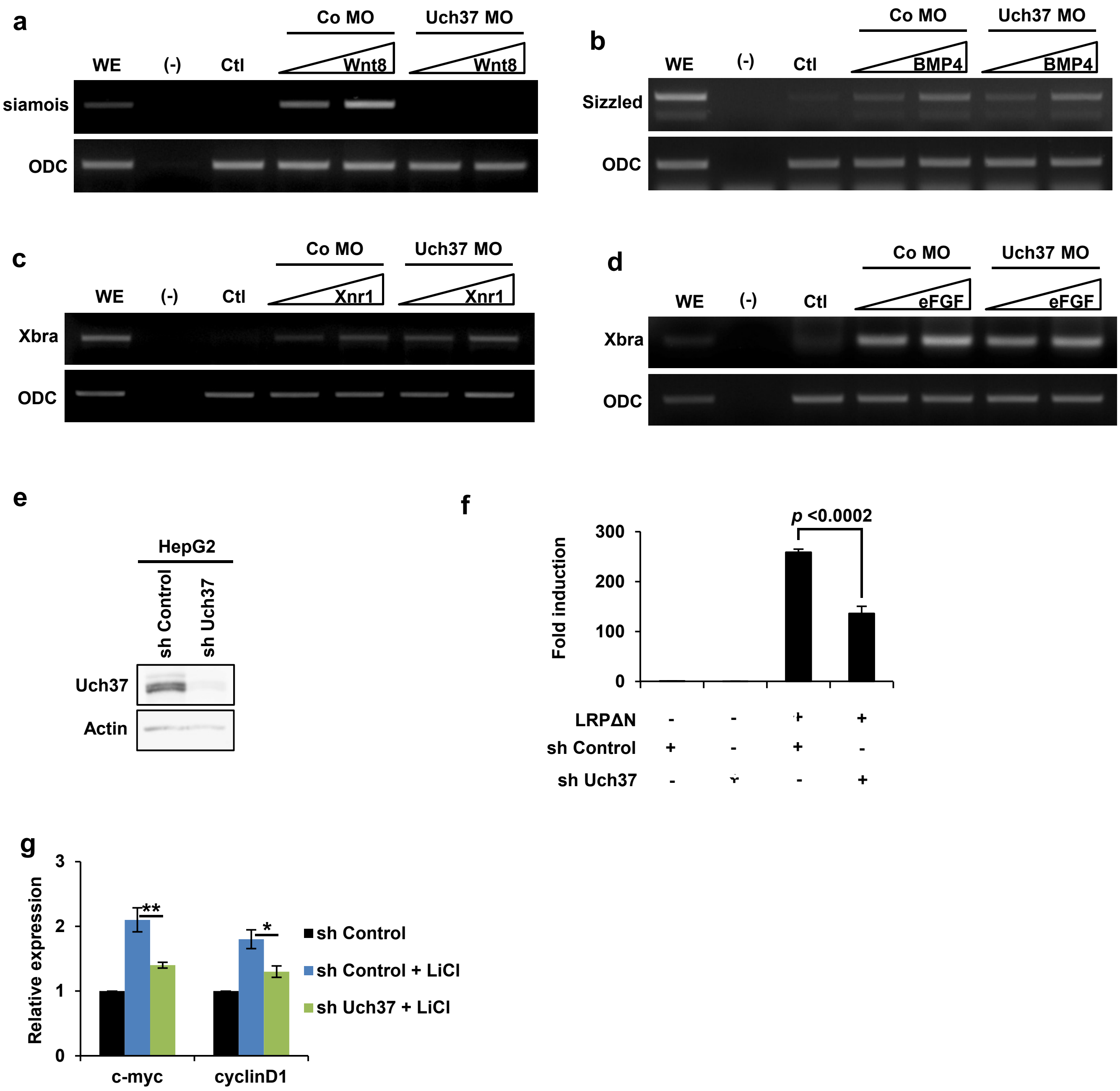

a
Co MO
Uch37 MO
WE
(-)
Ctl
Wnt8
Wnt8
siamois
ODC
b
Co MO
Uch37 MO
WE
(-)
Ctl
BMP4
BMP4
Sizzled
ODC
c
Co MO
Uch37 MO
WE
(-)
Ctl
Xnr1
Xnr1
Xbra
ODC
d
Co MO
Uch37 MO
WE
(-)
Ctl
eFGF
eFGF
Xbra
ODC
e
HepG2
sh Control
sh Uch37
Uch37
Actin
f
p <0.0002
300
200
100
0
Fold induction
LRPΔN
sh Control
sh Uch37
g
Relative expression
3
2
1
0
**
*
c-myc
cyclinD1
sh Control
sh Control + LiCl
sh Uch37 + LiCl

Figure S5

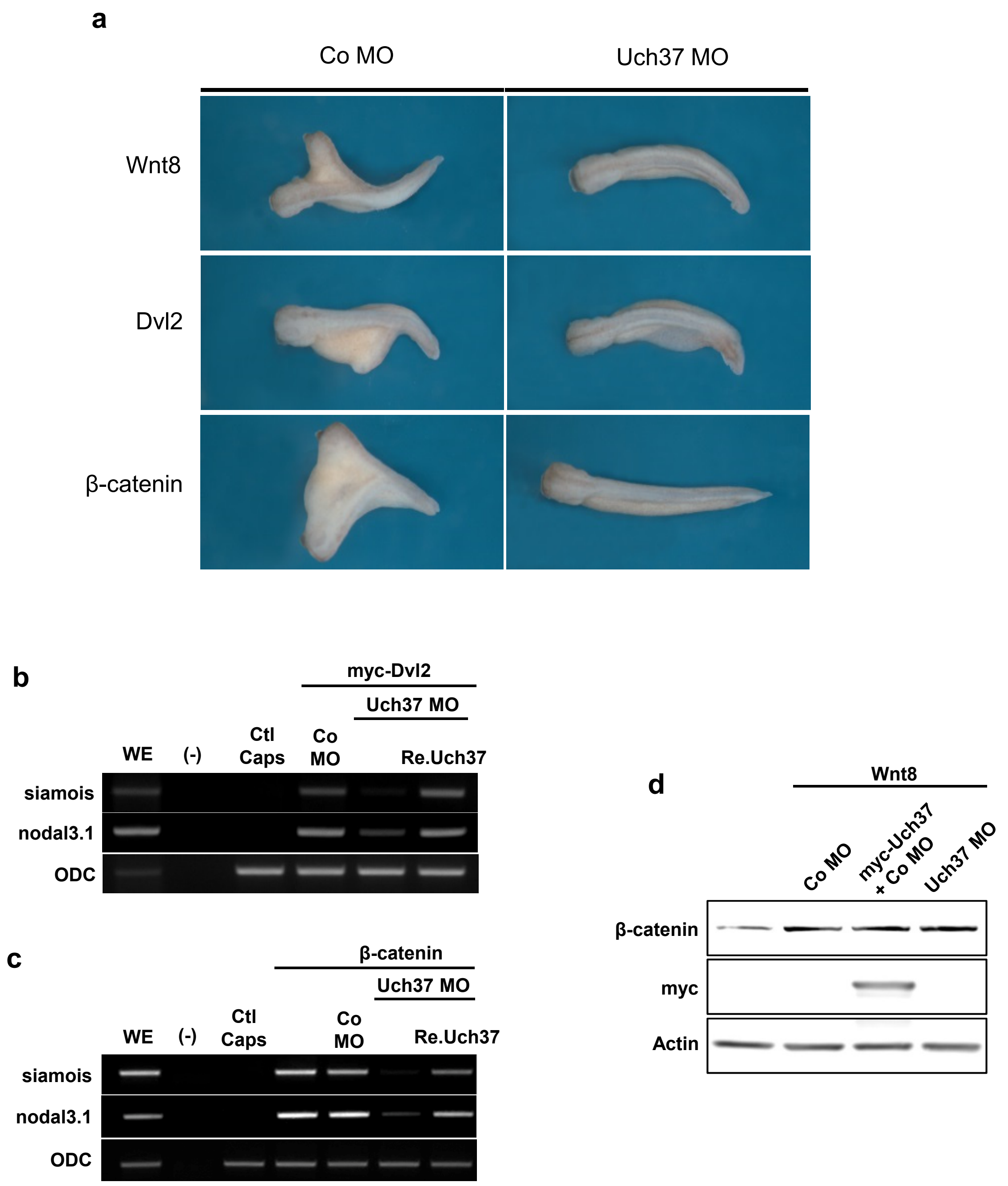

a
Co MO
Uch37 MO
Wnt8
Dvl2
β-catenin
b
myc-Dvl2
Uch37 MO
WE
(-)
Ctl Caps
Co MO
Re.Uch37
siamois
nodal3.1
ODC
c
β-catenin
Uch37 MO
WE
(-)
Ctl Caps
Co MO
Re.Uch37
siamois
nodal3.1
ODC
d
Wnt8
Co MO
myc-Uch37 + Co MO
Uch37 MO
β-catenin
myc
Actin

Figure S6

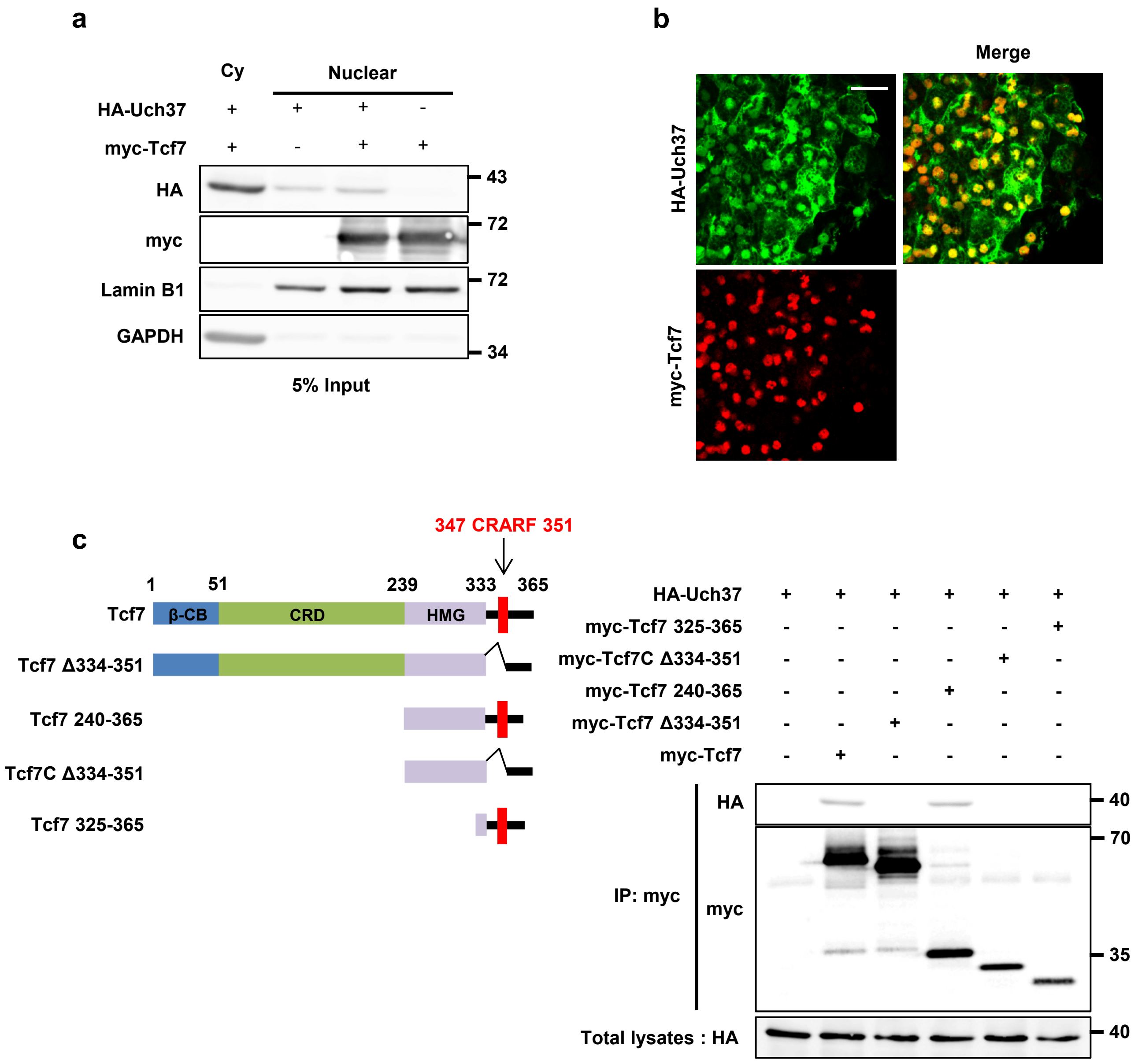
a
Cy
Nuclear
HA-Uch37 + + + -
myc-Tcf7 + - + +
HA
myc
Lamin B1
GAPDH
43
72
72
34
5% Input
b
Merge
HA-Uch37
myc-Tcf7
c
347 CRARF 351
1
51
239
333
365
Tcf7
β-CB
CRD
HMG
Tcf7 Δ334-351
Tcf7 240-365
Tcf7C Δ334-351
Tcf7 325-365
HA-Uch37 + + + + + +
myc-Tcf7 325-365 - - - - - +
myc-Tcf7C Δ334-351 - - - - + -
myc-Tcf7 240-365 - - - + - -
myc-Tcf7 Δ334-351 - - + - - -
myc-Tcf7 - + - - - -
HA
IP: myc
myc
Total lysates : HA
40
70
35
40

Figure S7

a

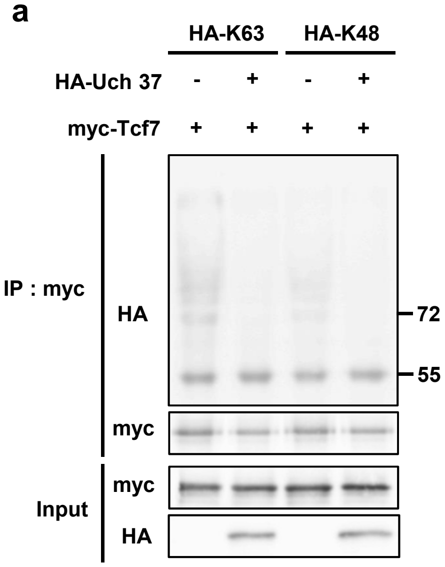

HA-K63
HA-K48
HA-Uch 37
- + - +
myc-Tcf7
+ + + +
IP : myc
HA
72
55
myc
Input
myc
HA

b

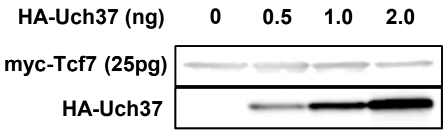

HA-Uch37 (ng)
0
0.5
1.0
2.0
myc-Tcf7 (25pg)
HA-Uch37

Figure S8

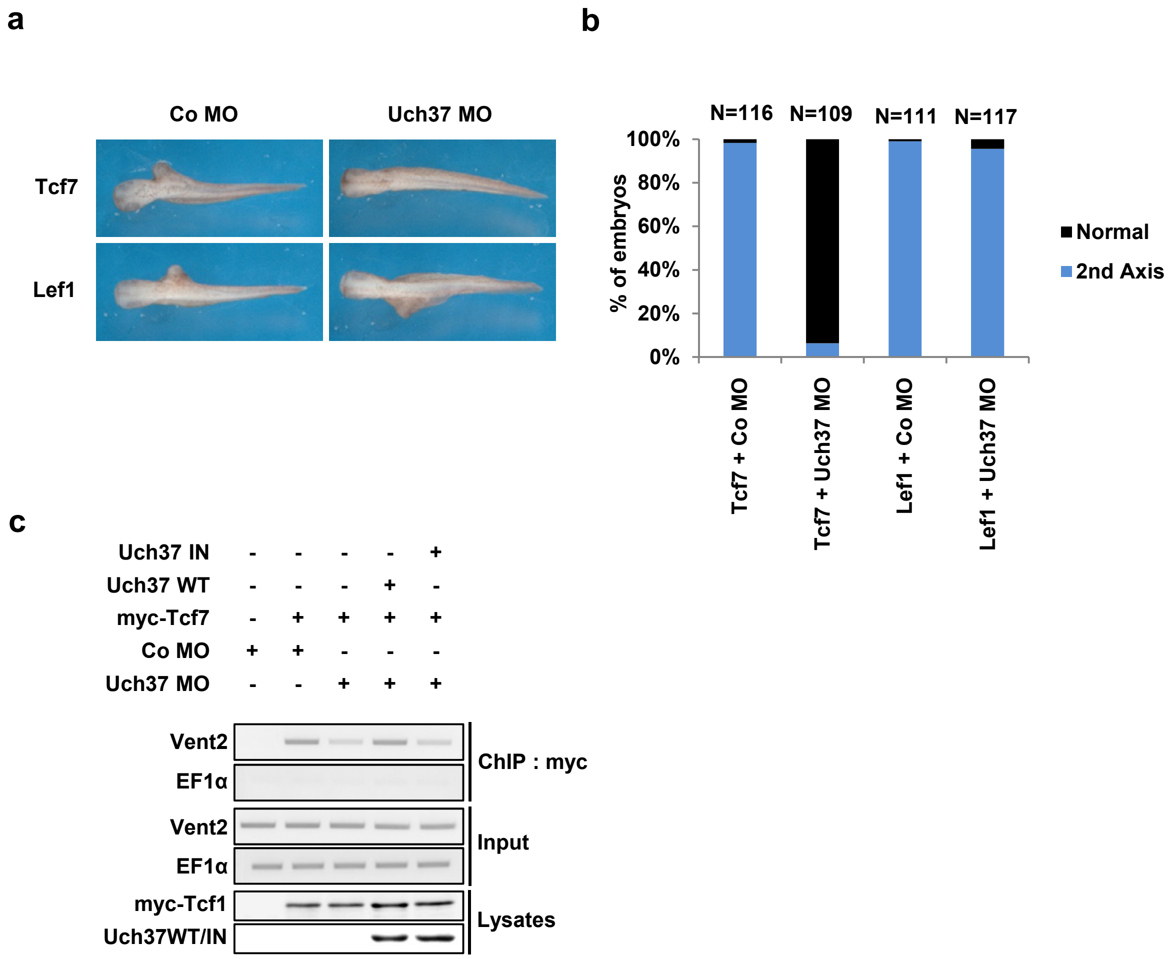

a
Co MO
Uch37 MO
Tcf7
Lef1
b
N=116
N=109
N=111
N=117
100%
80%
60%
40%
20%
0%
% of embryos
Normal
2nd Axis
Tcf7 + Co MO
Tcf7 + Uch37 MO
Lef1 + Co MO
Lef1 + Uch37 MO
c
Uch37 IN
Uch37 WT
myc-Tcf7
Co MO
Uch37 MO
Vent2
EF1α
ChIP : myc
Vent2
EF1α
Input
myc-Tcf1
Uch37WT/IN
Lysates

Figure S9

Figure 2a

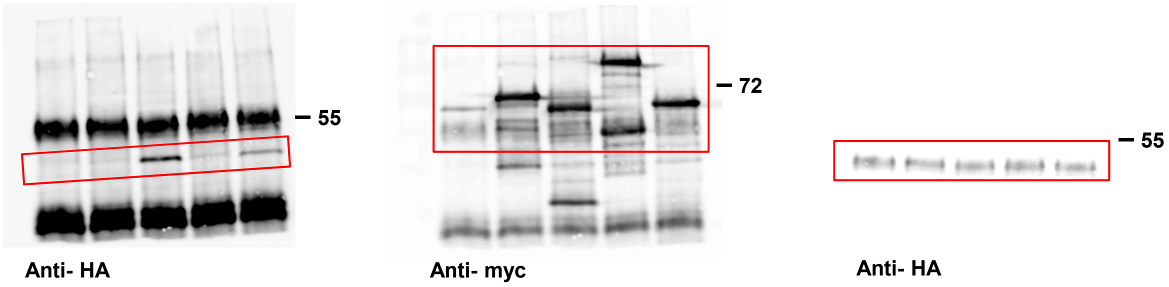

Figure 2b

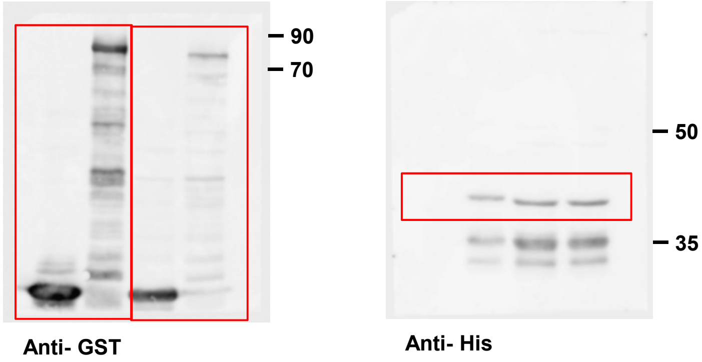

Figure 2c

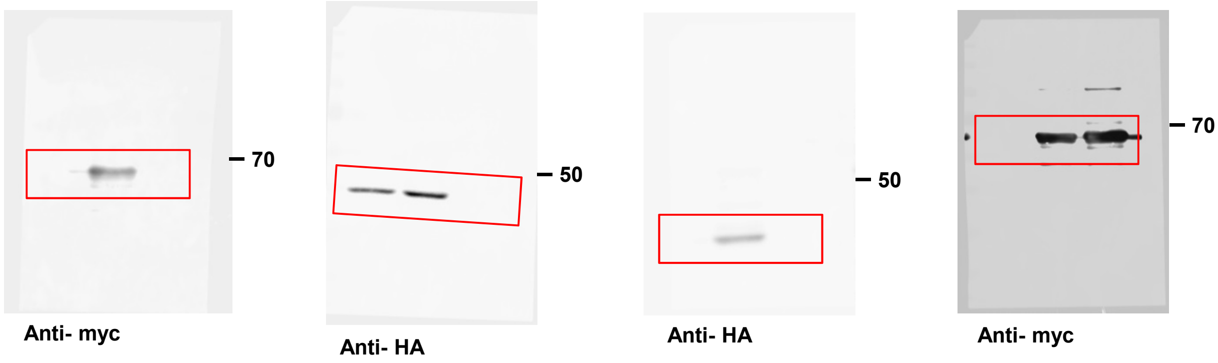

Figure 2d

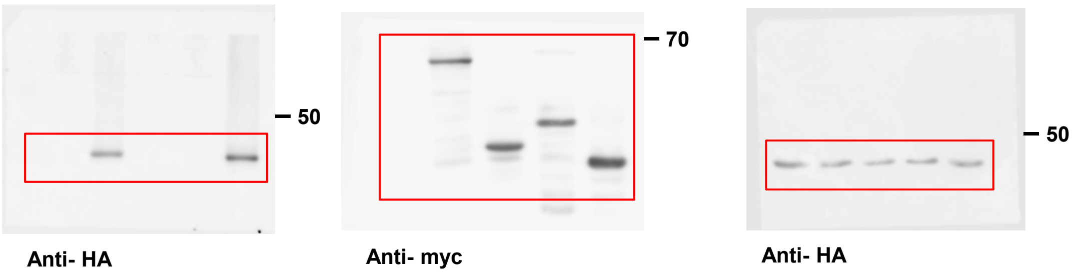

Figure S10

Figure 3a

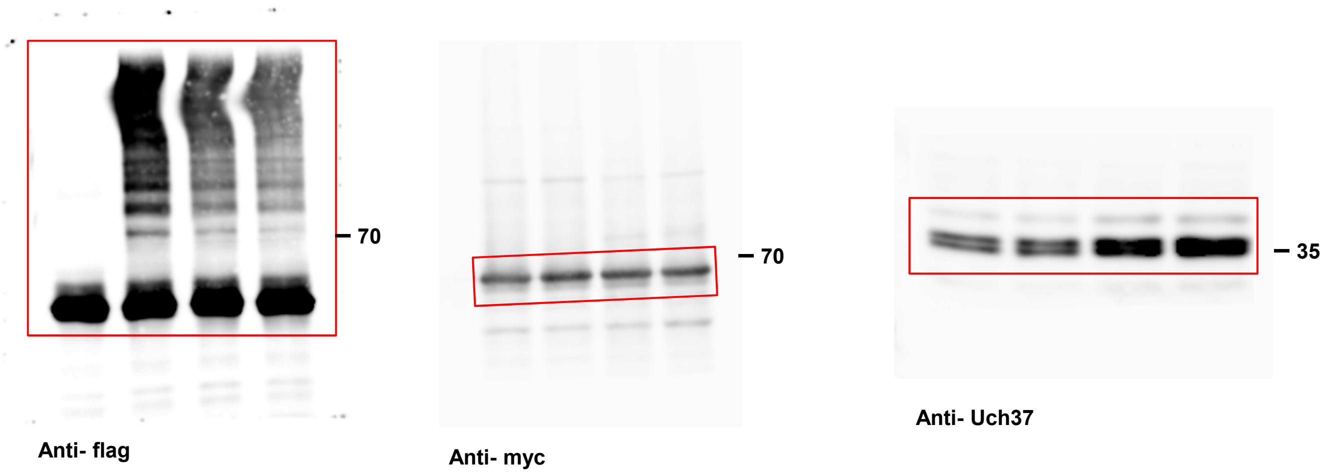

Figure 3b

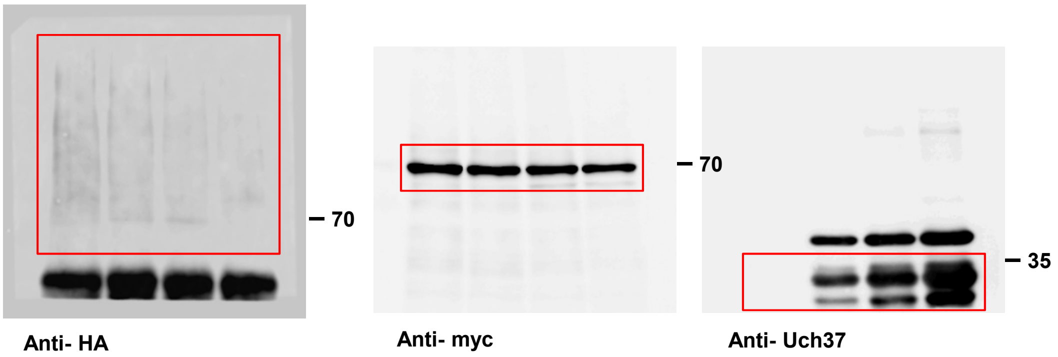

Figure 3c

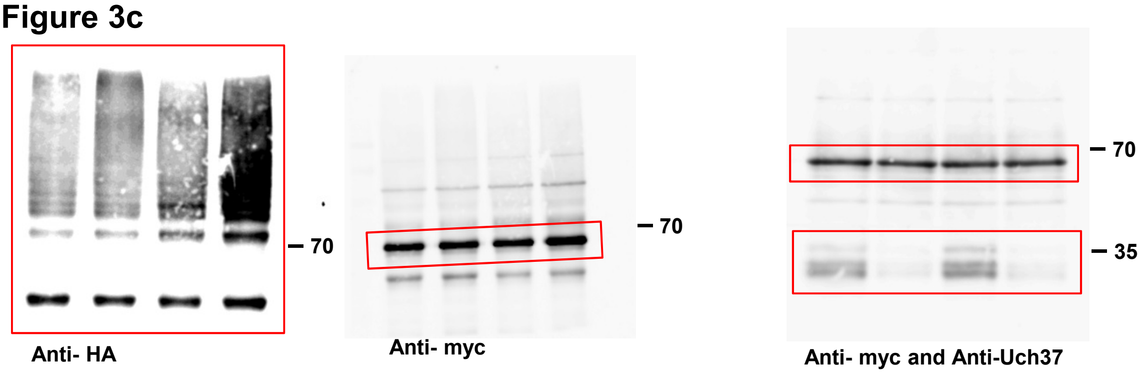

Figure S11

Figure 3e

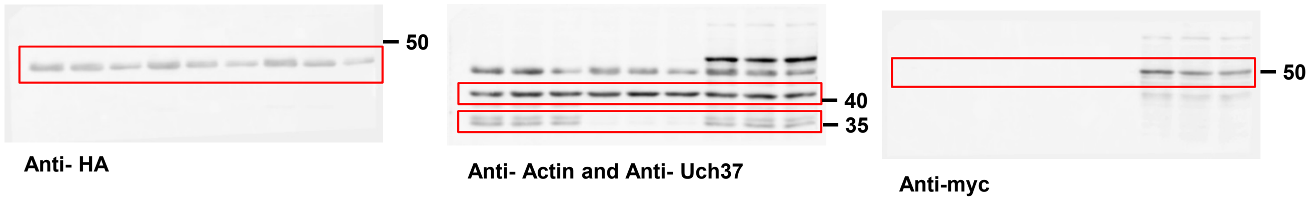

Figure 4d

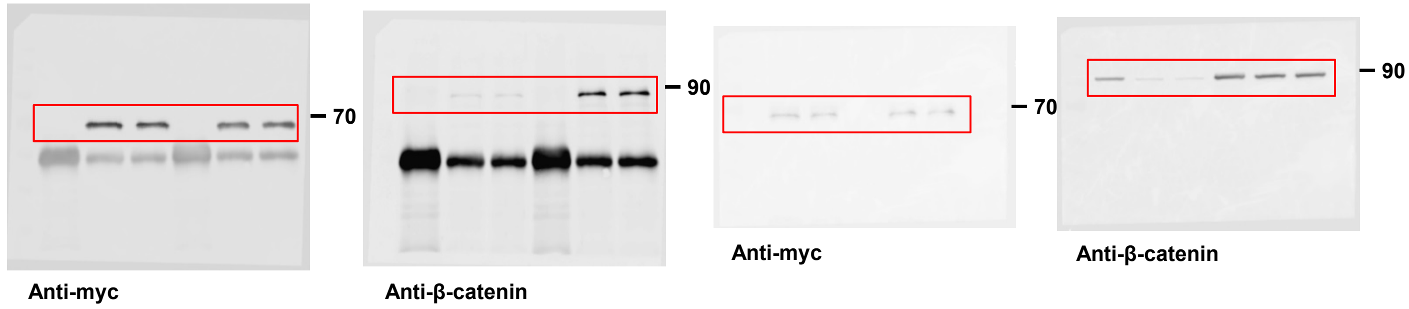

Figure 4e

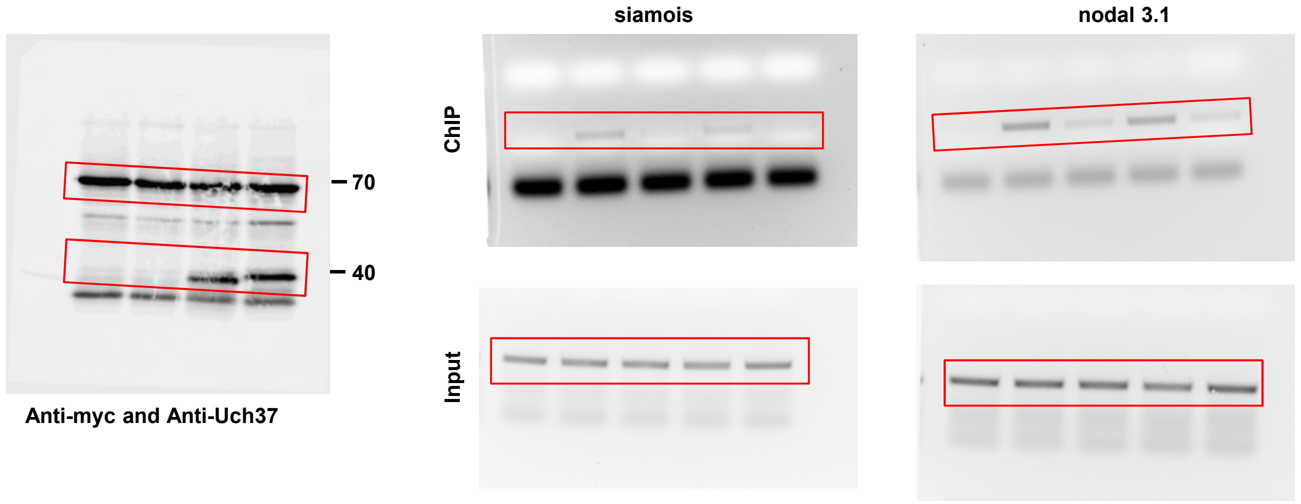

Figure S12

Figure 1b

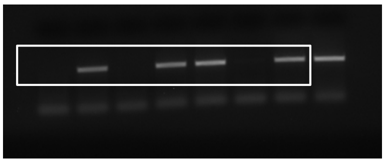

siamois

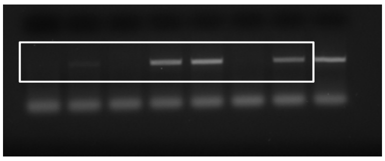

nodal 3.1

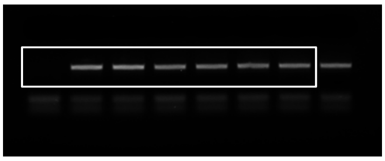

ODC

Figure 4a

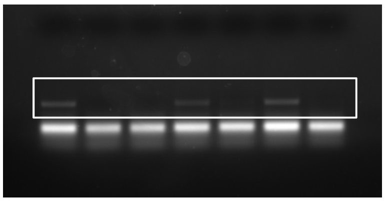

siamois

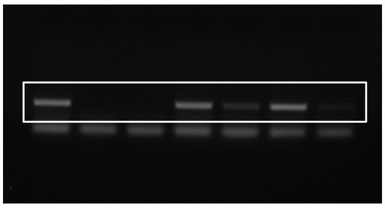

nodal 3.1

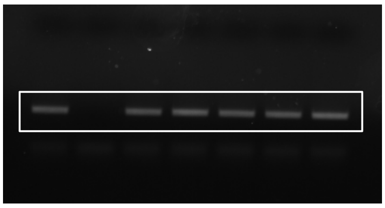

ODC

Figure 6c

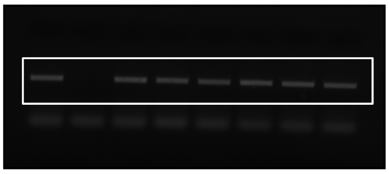

ODC

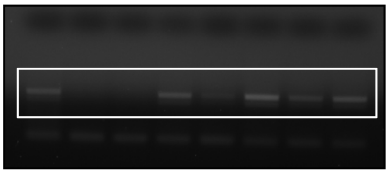

GSC

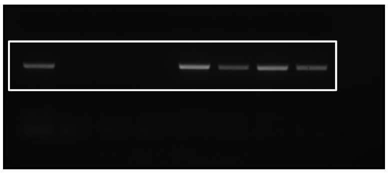

Xpo

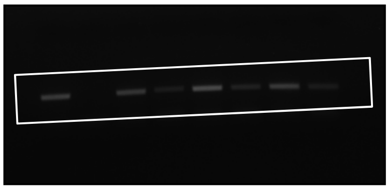

vent1

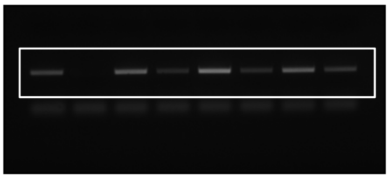

vent2

Table S1. Knockdown analysis (Supplementary Fig. S3b-d)

VMZ	1st			2nd			3rd		
	Co MO	Uch37 MO	Uch37 MO + Re.Uch37	Co MO	Uch37 MO	Uch37 MO + Re.Uch37	Co MO	Uch37 MO	Uch37 MO + Re.Uch37
N	37	42	39	42	36	29	35	33	41
Normal	37	7	16	42	4	10	35	2	18
Mild	0	21	19	0	19	16	0	19	16
Severe	0	14	4	0	13	3	0	12	7

DMZ	1st			2nd			3rd		
	Co MO	Uch37 MO	Uch37 MO + Re.Uch37	Co MO	Uch37 MO	Uch37 MO + Re.Uch37	Co MO	Uch37 MO	Uch37 MO + Re.Uch37
N	45	35	32	50	40	33	40	41	30
Normal	45	5	4	50	1	8	40	2	3
Mild	0	6	18	0	9	10	0	8	21
Severe	0	24	10	0	30	15	0	31	6

Table S2. Axis duplication assay (Fig. 1c, d)

	1st			2nd			3rd		
	Normal	Partial	Complete	Normal	Partial	Complete	Normal	Partial	Complete
Uninjected	40	0	0	36	0	0	60	0	0
Wnt8	2	32	0	3	37	0	10	30	0
Wnt8 + Uch37	2	2	34	0	5	27	1	0	48
Wnt8 + Uch37MO	40	0	0	38	0	0	54	0	0

Table S3. Axis duplication assay (Fig. 1g)

		1st		2nd		3rd	
		2nd Axis	Normal	2nd Axis	Normal	2nd Axis	Normal
Wnt8	Co MO	35	4	30	0	40	0
	Uch37 MO	0	33	1	34	0	37
Dvl2	Co MO	29	0	37	2	40	0
	Uch37 MO	3	34	6	29	4	36
ß-catenin	Co MO	28	0	35	0	40	0
	Uch37 MO	5	32	4	28	9	31

Table S4. Reduced ventrolateral mesoderm genes following depletion of Uch37 (Fig. 5a).

MyoD	1st				2nd				3rd			
	Co MO	Uch37 MO	Uch37 MO + Re.Uch37	Uch37 MO + Lef1	Co MO	Uch37 MO	Uch37 MO + Re.Uch37	Uch37 MO + Lef1	Co MO	Uch37 MO	Uch37 MO + Re.Uch37	Uch37 MO + Lef1
N	40	30	35	37	40	36	34	39	37	39	35	32
Normal	40	6	23	25	40	2	18	23	37	7	13	15
Reduced	0	24	12	12	0	34	16	16	0	32	22	17

Xpo	1st				2nd				3rd			
	Co MO	Uch37 MO	Uch37 MO + Re.Uch37	Uch37 MO + Lef1	Co MO	Uch37 MO	Uch37 MO + Re.Uch37	Uch37 MO + Lef1	Co MO	Uch37 MO	Uch37 MO + Re.Uch37	Uch37 MO + Lef1
N	37	41	42	39	41	38	36	34	35	37	38	35
Normal	37	5	24	25	41	6	22	19	35	3	18	21
Reduced	0	36	18	14	0	32	14	15	0	34	20	14

Vent1	1st				2nd				3rd			
	Co MO	Uch37 MO	Uch37 MO + Re.Uch37	Uch37 MO + Lef1	Co MO	Uch37 MO	Uch37 MO + Re.Uch37	Uch37 MO + Lef1	Co MO	Uch37 MO	Uch37 MO + Re.Uch37	Uch37 MO + Lef1
N	38	32	33	37	35	42	39	38	40	37	39	38
Normal	38	6	17	19	35	4	20	22	40	8	20	24
Reduced	0	26	16	18	0	38	19	16	0	29	19	14

Table S5. Uch37 MO inhibited promotion of the ventrolateral mesoderm genes by Wnt8 (Fig. 5b).

Vent1	1st			2nd			3rd		
	Co MO	Co MO + pCSKA Wnt8	Uch37 MO + pCSKA Wnt8	Co MO	Co MO + pCSKA Wnt8	Uch37 MO + pCSKA Wnt8	Co MO	Co MO + pCSKA Wnt8	Uch37 MO + pCSKA Wnt8
N	30	42	51	29	37	34	30	33	31
Normal	30	7	4	29	10	5	30	13	0
Promoted	0	35	6	0	27	9	0	20	11
Reduced	0	0	41	0	0	20	0	0	20

Xpo	1st			2nd			3rd		
	Co MO	Co MO + pCSKA Wnt8	Uch37 MO + pCSKA Wnt8	Co MO	Co MO + pCSKA Wnt8	Uch37 MO + pCSKA Wnt8	Co MO	Co MO + pCSKA Wnt8	Uch37 MO + pCSKA Wnt8
N	30	34	42	26	30	37	30	34	29
Normal	30	6	10	26	10	0	30	16	1
Promoted	0	28	2	0	20	12	0	18	0
Reduced	0	0	30	0	0	25	0	0	28

Figure S1 Uch37 is highly conserved in vertebrate species including human, mouse and *Xenopus*. Comparison of Uch37 peptides across vertebrate species. The most conserved residues are indicated by the shaded background. Distinct domains are indicated with colored lines (red, catalytic domain; blue, C-terminal extension).

Figure S2 Embryonic expression of Uch37. (a) Expression of Uch37 in *Xenopus* embryo was analyzed by RT-PCR. ODC, ornithine decarboxylase loading control; (-), -RT; egg, unfertilized; St, Nieuwkoop and Faber (NF) developmental stage. (b) Spatial expression of Uch37 from cleavage to gastrula stage. Red box indicates the magnified mesoderm region. (c) Expression of Uch37 in various dissected tissues at stage 10.5. WE, whole embryo; AC, animal cap; DMZ, dorsal marginal zone; VMZ, ventral marginal zone; Chordin, dorsal marker; Msx1, ventral marker; Xbra, pan-mesodermal marker.

Figure S3 Phenotype of Uch37 morphant. (a) Uch37 MO specifically inhibits the translation of C-terminally HA-tagged Uch37 mRNA (1ng), but not MO-resistant Uch37 (Re.Uch37) mRNA (1ng) and human Uch37 mRNA (1ng). Four-cell stage embryos were injected in animal blastomeres with indicated reagents. Embryos were cultured to stage 10.5 and then subjected to western blotting. (b) Phenotypes of Uch37-depleted embryos. Embryos were injected at the four-cell stage with the indicated reagents (40 ng Co MO; 40 ng Uch37 MO; 2ng Re.Uch37) ventrally (b, left) or dorsally (b, right) and cultured to tadpole stage. (c, d) Morphant phenotypes were tabulated.

Figure S4 Uch37 specifically regulates Wnt signaling. (a-d) RT-PCR analysis using animal cap explants. Co MO (20ng) or Uch37 MO (20ng) were respectably or co-

injected with indicated mRNAs encoding Wnt8 (10pg and 20pg), BMP4 (50pg and 100pg), Xnr1 (50pg and 100pg), or eFGF (100pg and 200pg) in animal blastomeres of four-cell stage embryo. Animal cap explants were dissected at stage 9 and cultured until stage 11. siamois, Wnt-target gene (a); Sizzled, Bmp4-target gene (b); Xbra, target gene of Xnr1 (c) and eFGF (d). (e) Short hairpin RNA (shRNA) of Uch37 efficiently reduced levels of Uch37 protein in HepG2 cells. (f) TOPflash assay in HepG2 cells. Reporter constructs were transfected alone or co-transfected with constitutively active LRP6 (LRP6ΔN) into stable cells (sh Control and sh Uch37). 48h after transfection, luciferase activity was measured. (g) qPCR analysis for the expression of Wnt-target genes (c-myc and cyclinD1) in stable HepG2 cells (sh Control or sh Uch37) treated with or without LiCl (25mM). The quantities of indicated mRNA were normalized by β-actin. Data represent average values from three independent experiments performed. Error bars indicate standard deviations of triplicate. *, $p < 0.005$; **, $p < 0.001$ (two-tailed Student's ttest).

Figure S5 Uch37 promotes Wnt signaling downstream of ß-catenin destruction complex. (a) Result of axis duplication assay in Fig 1g. (b, c) RT-PCR analysis using animal cap explants. Embryos were animally injected at four-cell stage. Animal cap explants were isolated at stage 9 and cultured until stage 11. Injected reagents are as follows, Co MO (20ng), Uch37 MO (20ng), myc-Dvl2 mRNA (200pg), ß-catenin mRNA (25pg), Re.Uch37 (1ng). (d) Western blot analysis using animal cap explants. Embryos were animally injected at two-cell stage. Animal cap explants were dissected at stage 9 and cultured until stage 11. Injected reagents are as follows, Co MO (40ng), Uch37 MO (40ng), Wnt8 mRNA (20pg), myc-Uch37 mRNA (1ng).

Figure S6 Uch37 interacts with Tcf7 in nucleus of *Xenopus* gastrula embryo.

(a) Fractionated lysates from *Xenopus* gastrula embryos were used as expression input for Co-IP analysis in Fig 1A. (b) Uch37 is co-localized with Tcf7 in nucleus**.** Four-cell stage embryos were animally co-injected with HA-Uch37 (1 ng) and myc-Tcf7 (50pg). Animal cap explants were isolated at stage 10 and fixed with 4% paraformaldehyde for 2h. HA-Uch37 and myc-Tcf7 were immunostained with anti-HA (mouse, santacruz; 2nd antibody, Alexa-488, invitrogen) and anti-myc antibodies (rabbit santacruz; 2nd antibody, Alexa-594, invitrogen). Scale bar (white line) indicates 50μm. (c) Co-IP assay using HEK293FT cells, HA-Uch37 and indicated myc-tagged truncated mutants of Tcf7 were transfected. Cell lysates were immunoprecipitated with anti-myc antibody. Truncated mutants of Tcf7 are depicted on the left.

Figure S7 Uch37 is not involved in stabilization of Tcf7 protein. (a) *In vivo* ubiquitination assay using HEK293FT cells. Ectopically expressed HA-Uch37 reduced both K48-and K63-mediated polyubiquitin chains from Tcf7. (b) Western blot analysis. Embryos were animally injected at two-cell stage with indicated amount of mRNAs. Animal cap explants were isolated at stage 9 and cultured until stage 11, and then subjected to immunoblotting.

Figure S8 Uch37 MO specifically blocks Tcf7-mediated Wnt activity and Uch37 is required for Tcf7 binding to promoter of Vent2. (a) Axis duplication assay. Four-cell stage embryos were injected in one ventrovegetal blastomere with indicated

reagents (400pg Tcf7 mRNA; 400pg Lef1; 40ng Co MO; 40ng Uch37 MO). (b) Quantified result of a. (c) ChIP assay using *Xenopus* embryos (stage 11). 70 embryos were injected at two-cell stage as indicated (25pg myc-Tcf7 mRNA; 1ng wild type of Uch37 (WT); 1ng catalytically inactive Uch37 (IN); 40ng Co MO; 40ng Uch37 MO). Lysates were precipitated with anti-myc antibody. Precipitated Wnt target DNAs were analyzed by PCR. EF1α was used as a control for specificity.

Figure S9-S12 Full-length images of gels and blots from the main figures.
